# Designing fisetin nanocrystals for enhanced *in cellulo* anti-angiogenic and anticancer efficacy^☆^

**DOI:** 10.1016/j.ijpx.2022.100138

**Published:** 2022-11-09

**Authors:** Panpan Ma, Johanne Seguin, Nhu Ky Ly, Luis Castillo Henríquez, Eva Plansart, Karim Hammad, Rabah Gahoual, Hélène Dhôtel, Charlotte Izabelle, Bruno Saubamea, Cyrille Richard, Virginie Escriou, Nathalie Mignet, Yohann Corvis

**Affiliations:** a Université Paris Cité, CNRS, INSERM, UTCBS, Chemical and Biological Technologies for Health Group (utcbs.u-paris.fr), 75006 Paris, France; b Université Paris Cité, CNRS, CiTCoM, 75006 Paris, France; c Université Paris Cité, UAR3612 CNRS, US25 INSERM, Cellular and Molecular Imaging Facility, 75006 Paris, France

**Keywords:** Flavone derivative, Solvent/antisolvent nanoprecipitation, Crystalline nanosuspension, Sustained drug release, *In vitro* evaluation

## Abstract

We report the formulation, characterization, colloidal stability, and *in vitro* efficiency of Fisetin nanocrystals stabilized by poloxamer P407. Such nanocrystals present a nanometer scale (148.6 ± 1.1 nm) and a high homogeneity (polydispersity index of 0.17 ± 0.01), with a production yield of 97.0 ± 2.5%. The engineered formulations of nanocrystals suspension (pH of 7.4 ± 0.1), stabilized *via* steric repulsion, are stable for several days in aqueous environment (Milli Q water, NaCl 10 mM or mannitol 5% w/v), for few days in HEPES buffered saline (HBS) (20 / 150 mM) under sink conditions, and in culture medium. After freeze drying in 5% w/v mannitol, the nanocrystal formulations can be stored at −80 °C for at least 120 days. Drug release experiments displayed a 98.7 ± 5.1% cumulative release over 3 days in HBS. Compared to the free drug, the nanocrystal formulations showed an improved cytotoxicity highlighted by the decrease of the half maximal inhibitory concentration for both murine Lewis lung carcinoma (3LL) and human endothelial (EA.hy926) cell lines. In addition, after incubation with Fisetin nanosuspensions, significant changes in the cell morphology for both cell lines were observed, showing an improved anti-angiogenic effect of nanocrystals formulation compared to the free drug. Overall, Fisetin formulated as nanocrystals showed enhanced biopharmaceutical properties and *in vitro* activity, offering a wide range of indications for challenging applications in the clinic.

## Graphical abstract

**Figure:**
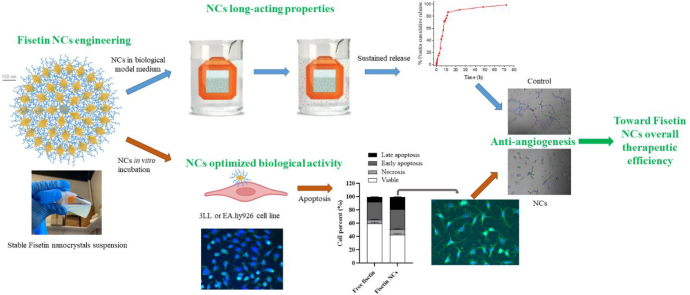

## 1 Introduction

Due to patient compliance, cost-effectiveness, ease of administration, and stability issues, 80% of the marketed drugs are suitable for formulation in solid dosage forms (Dhapte and Mehta, 2015; Vieth et al., 2004; Couillaud et al., 2019). However, 40% the marketed drugs and 70 to 90% of those still under investigation by the regulatory authorities exhibit low solubility (Ting et al., 2018; Nikolakakis and Partheniadis, 2017). Therefore, enhancing the solubility of poorly water-soluble drugs is a major challenge. In order to transfer a drug formulation from R&D to the clinic, its circumstantial study should be conducted notably by studying physical, chemical, biopharmaceutical, biocompatibility, and bioavailability properties (Brittain, 1999; Couillaud et al., 2019). To date, many approaches have been developed to conquer the bioavailability limitations of poorly water-soluble drugs (Cooper, 2010). With the growth of nanotechnology in recent years, nanocrystals (NCs) formulations, which consist in using the active pharmaceutical ingredient (API) stabilized by surface-active agents, have been notably developed to improve loading efficiency, bypass the use of drug carriers, and overall increase the bioavailability of the API. In addition, compared to solubilized liquid dosage forms, the NCs formulations can be functionalized to target specific tissues, while preserving sustained release properties, and thus overall modify the therapeutic impact of the drug (Couillaud et al., 2019; Deguchi et al., 2021; Fan et al., 2022; Ige et al., 2013; McGuckin et al., 2022; Müller et al., 2011). Consequently, NCs can be considered carrier-free colloidal assemblies with size in the submicron scale for drug delivery purposes (Junghanns and Müller, 2008) and administrated through all routes, mainly due to the nanometric particle size (Chen et al., 2015). Hence, the emergence of NCs for drug development in the last 13 years highlights the importance of formulating new nanomaterials for improved therapeutic efficiency. Furthermore, it has been proven that poloxamer P407, an FDA-approved polymer as a solubilizing or stabilizing agent, can inhibit the P-glycoprotein pumps, which contribute to multiple drug resistance (Batrakova et al., 1999; Saxena and Hussain, 2012). Consequently, the engineering of Fisetin NCs stabilized by P407 copolymer reinforces the legitimacy of drug NCs as potent forthcoming delivery systems for nanomedicine and opens the way to their potential marketability (Lepeltier et al., 2020; Xiang et al., 2022).

**Scheme 1:**
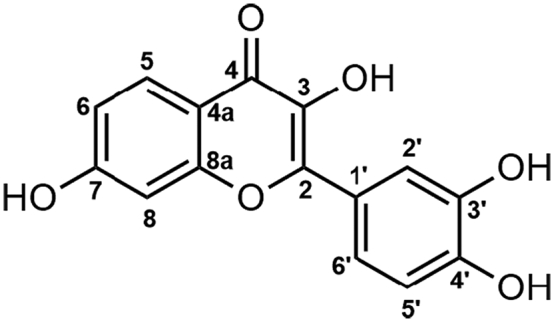
Chemical structure of Fisetin, with the IUPAC numbering (Rauter et al., 2018).

3,3′,4′,7-tetrahydroxyflavone (Scheme 1), otherwise known as Fisetin is a natural bioactive diphenylpropane flavone abundantly found in different kinds of nuts, wine, fruits, vegetables, and teas (Arai et al., 2000; Grynkiewicz and Demchuk, 2019; Kimira et al., 1998). This flavonoid with anti-angiogenic, anti-inflammatory, anti-tumorigenic, anti-oxidant, and/or neuroprotective biological effects (Ahmad et al., 2021; Bothiraja et al., 2014; Imran et al., 2021; Park et al., 2007; Woodman and Chan, 2004), is therefore considered as a senotherapeutic agent (Elsallabi et al., 2022). Several studies proved that Fisetin inhibits biological pathways such as androgen receptor signaling, DNA topoisomerases I and II, urokinase, and cyclin-dependent kinases activities (Afroze et al., 2022; Constantinou et al., 1995; Elsallabi et al., 2022; Khan et al., 2008; Olaharski et al., 2005; Sung et al., 2007; Syed et al., 2016). This API has also demonstrated a synergistic interaction with RNA Pol I inhibitor, BMH-21, and displays significant anticancer effects by reducing lung colonization of breast cancer cells (Kammerud et al., 2021). In addition, in the fight against the COVID-19 pandemic, Fisetin has been considered for treating skilled nursing facility residents with long-term effect symptoms (Verdoorn et al., 2021).

However, clinical application of Fisetin has been seriously limited due to its low aqueous solubility (9.55–10.45 μg.mL^−1^ at 37 °C) (Elsallabi et al., 2022; Jeong et al., 2013; Moon et al., 2022) and consequent poor bioavailability, which may require frequent administration to patients (Wissler Gerdes et al., 2021; Khozooei et al., 2022; Mehta et al., 2018). This API has been reported to exhibit a higher solubility in other polar solvents, such as ethanol (4.35 mg.mL^−1^) and methanol (4.65 mg.mL^−1^), as well as mixtures of methanol/chloroform (50/50% v/v, 9.75 mg.mL^−1^) (Mignet et al., 2012) and ethanol/water (50/50% v/v, 2.89 mg.mL^−1^) (Elsallabi et al., 2022). At physiological pH, the molecule is mostly in its unionized form (pKa_1_ = 7.27; pKa_2_ = 9.44), which allows it to passively pass through cell membranes (Naeimi and Alizadeh, 2017). However, its low solubility, high lipophilicity (log *P* = 2.2), and low intrinsic dissolution rate explain the relatively low oral bioavailability (44.1%) that limits its therapeutic potential when administered to patients in doses up to 20 mg.kg^−1^ (Grynkiewicz and Demchuk, 2019). In addition, Fisetin oral doses are rapidly degraded in the gastrointestinal tract, as well as undergo hepatic-first pass metabolism and P-glycoprotein-mediated efflux (Kadari et al., 2017).

Previous studies from our lab have shown that Fisetin improves anticancer and anti-angiogenic activities *in vitro* and *in vivo* (Touil et al., 2011). Based on these results, our laboratory has developed several formulations to solubilize and deliver Fisetin, *i.e.*, emulsions (Ragelle et al., 2012), spherulites (Crauste-Manciet et al., 2013), and liposomal forms (Mignet et al., 2012; Renault-Mahieux et al., 2021; Seguin et al., 2013). Among them, liposomal formulations encapsulating Fisetin or co-encapsulating Fisetin and Cisplatin were prepared and evaluated by *in vitro* cytotoxicity experiments on the Lewis lung carcinoma (3LL) and human umbilical vein endothelial (EA.hy926) cell lines, as well as human glioblastoma cell line U-87 MG (Mignet et al., 2012; Renault-Mahieux et al., 2021). The results were promising enough to go further with *in vivo* evaluation in mice (Seguin et al., 2013). However, all formulations suffered from both non-optimal encapsulation rate at d0 (mean yield of 58%) and loss of Fisetin with aging (loss of 25% and 50% of the d0 content after 10 and 59 days, respectively), limiting further therapeutic use.

Therefore, in the present study, we propose to adapt an original technique recently developed in our lab, based on the solvent/antisolvent nanoprecipitation of an API stabilized by poloxamer P407 (Martin et al., 2020a), to prepare Fisetin NCs with improved encapsulation rate and loading content.

Fisetin NCs have been formulated and characterized using nuclear magnetic resonance (NMR) spectroscopy, mass spectrometry (MS), dynamic light scattering (DLS), classic and cryogenic transmission electron microscopy (TEM and cryo-TEM, respectively), scanning electron microscopy (SEM), confocal laser scanning microscopy (CLSM), Fourier transform Infra-Red (FTIR) spectroscopy, fluorescence spectroscopy, and high-performance liquid chromatography (HPLC). In addition, drug release profiles in a biological medium model were performed by dialysis. *In vitro* anticancer and anti-angiogenic properties of the Fisetin NCs have been further assessed *via* morphology/apoptosis, and capillary tube formation evaluations, respectively. The obtained results represent a step towards NCs production and therapeutic application.

## 2 Materials and methods

### 2.1 Materials

Fisetin (Mw = 286.24 g.mol^−1^, CAS number 528–48-3, batch N1032, 98% purity) was purchased from Shanghai FWD Chemicals Limited (China) and Ph. Eur. Pluronic 407 (mean Mw = 13,429.2 g.mol^−1^, determined by mass spectroscopy; data not shown) was purchased from BASF (Ludwigshafen, Germany). No further purification step was done for the NCs preparation. Methanol (MeOH) was purchased from Thermo Fisher Scientific Inc. (Waltham, Massachusetts, United States). Deionized water (by Milli-Q, filtered through a 0.2 μm membrane) was used for the present study. A 10-kDa regenerated cellulose membrane (Merck, Darmstadt, Germany) was used for filtrations prior to fluorescence spectroscopy experiments.

### 2.2 Nanocrystals preparation

**Fig. 1:**
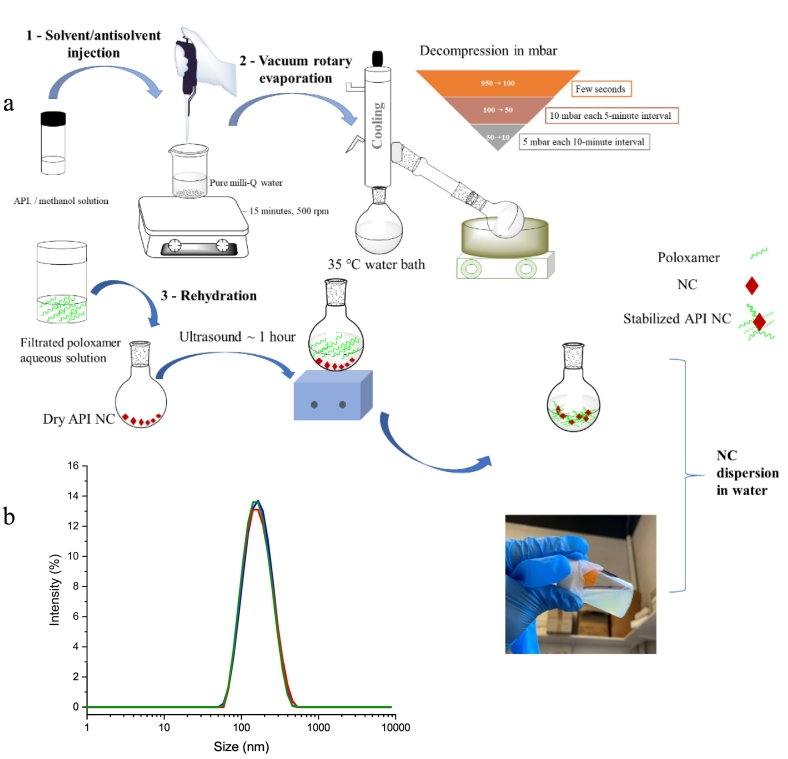
(a) Schematic representation of optimized API nanocrystal formulations process using a bottom-up technology. (b) Size distribution of Fisetin NCs obtained by DLS.

Fisetin NCs were prepared in lucifungal conditions by the solvent/antisolvent precipitation method, a bottom-up technology (Fig. 1a). Based on our previous results (Martin et al., 2020b), the optimal reaction parameters were selected, such as the nature of the solvent, the solvent/antisolvent ratio, the evaporation rate, and the surfactant concentration. Solvent and antisolvent were chosen based on solubility studies of Fisetin in different media. Water was used as antisolvent regarding Fisetin solubility (up to 10.45 μg.mL^−1^ in water at 37 °C and logP = 2.2) (Elsallabi et al., 2022), while MeOH was used as the solvent. Based on our patent (Martin et al., 2020a), the solvent/antisolvent volume ratio was established at 1.5:10, and the API/stabilizer mass ratio at 1:2. Poloxamer P407 was chosen to inhibit the crystal growth, allowing to engineer Fisetin NCs. Briefly, 2.5 mg of Fisetin were first completely dissolved in 1.5 mL of MeOH using ultrasonication for 10 min, and then added dropwise into 10 mL of water under agitation at 500 rpm. After 15 min of agitation, the solubilized drug was precipitated by evaporating the solvent-antisolvent azeotrope mixture through controlled decompression stages using a vacuum rotavapor (Buchi R-114, Flawil, Switzerland). The resulting powder was kept in a dry, lucifungal environment. Then, the optimal proportion of poloxamer P407 aqueous solution (0.083% w/v, filtered through a 0.22 μm Minisart® syringe filter) was added to the dry powder for hydration, and the suspension was subjected to 1-h sonication. Ice was added in the ultrasonic bath to prevent high temperature, as poloxamer P407 is a temperature-sensitive polymer. Ultrasonic treatment can control crystallization kinetics *via* an increase of the mixing, the nucleation rate, and the limitation of particle growth and agglomeration (Belca et al., 2019; Chang et al., 2015; Gielen et al., 2017; Sinha et al., 2013; Thakor et al., 2020). The ultrasonic energy was promoted by an ultrasonic bath (M1800-E, 40 kHz, Branson Ultrasonics™ CPX-952-136R). No further purification steps were required since the nanocrystals were directly obtained from the pure compound with no degradation of the API during the nanoprecipitation process as discussed in the Results section.

### 2.3 Storage of the Fisetin NCs samples

All the formulated NCs suspensions were kept in a glass vial, in a lucifungal environment at 5 °C. We have also shown that the NCs may be prepared after storage of the powder several days at room temperature (RT) in lucifungal conditions prior to the rehydration process.

### 2.4 Solid state preparation of NCs suspensions

In order to obtain the NCs in a solid state for SEM experiments, the NCs aqueous suspensions were dried by using a vacuum rotavapor (Buchi R-114, Flawil, Switzerland) with controlled decompression stages. Subsequently, the resulting powders were then used as-is.

### 2.5 NCs size, polydispersity, zeta potential, and morphology

The size of the NCs was determined from the translational diffusion coefficient by using the Stokes-Einstein formula (Makuch et al., 2020) at 25 °C by dynamic light scattering (DLS, Nano ZS Malvern) and Vasco Nano-Kin (France). From the second-order correlation function (correlogram) allowing to measure the degree of non-randomness in an apparent random data set, it is possible to determine the hydrodynamic diameter (mean size) and the polydispersity index (PDI) of particles dispersed in a given continuous medium. After 1 min sonication, the size measurement of NCs was performed as follows: 1 mL of the Fisetin NCs mother suspension (0.4 mg.mL^−1^) was transferred into a ZEN0040 cuvette and introduced in the DLS device (λ = 633 nm, scattering angle: 173 deg., fixed position of laser: 3 mm). As far as the Vasco Nano-Kin experiments are concerned, after laser alignment, Fisetin NCs were directly tested in the glass container without any transfer into a specific cuvette, allowing real-time measurement (λ = 635 nm, scattering angle: 170 deg.). For the zeta potential analysis, 10 μL of sodium chloride solution (NaCl, 1.01 M) was added to 1 mL of Fisetin NCs suspension (final NaCl concentration: 10 mM) before sample introduction into a DTS1070 cuvette. Each measurement was carried out in triplicate at 25 °C.

Stability of the Fisetin NCs was assessed in cell culture medium, *i.e.* Dulbecco's Modified Eagle Medium (DMEM, No31966021, Gibco, Paisley, United Kingdom) containing 10% fetal bovine serum (FBS, F7524, Sigma-Aldrich, Missouri, United States), and 1% penicillin-streptomycin (10,000 units per mL, and 10 mg.mL^−1^, respectively, No15140122, Gibco, Paisley, United Kingdom). For that purpose, 1 mL of Fisetin/P407 NCs suspension was added to 6 mL of DMEM complete medium. The resulting solution was sonicated for 1 min, and 1 mL was transferred into the DLS cuvette for total derived count rate measurement by the DLS device. Since the measurements were performed over time, between each measurement, the 1 mL solution of the cuvette was transferred back in the NCs/cell culture medium and kept at 5 °C. Finally, the resulting solution was sonicated for 1 min before each DLS measurement over time.

The morphology of Fisetin NCs was investigated by SEM. For that purpose, raw Fisetin powder and formulated Fisetin/P407 NCs powder (obtained after removing the water as described in the previous section) were placed on a double-sided tape, sputtered with gold/palladium, and observed under high vacuum at 15 kV (SEM 6510LV, Jeol, Croissy-sur-Seine, France).

Additionally, the structure of Fisetin nanosuspensions was evaluated by TEM and cryo-TEM. For TEM, a 200-mesh carbon formvar copper grid was covered with one drop (10 μL) of the Fisetin NCs suspension, blotted and dried at RT for two hours. For cryo-TEM, samples were prepared using an EM-GP2 cryoplunger (Leica Microsystems, Nanterre, France) with an environmental chamber set at 15 °C and 95% humidity. A 4 μL aliquot of the Fisetin NCs suspension was applied on a glow discharged 300 mesh copper grid covered with a lacey carbon film (Agar Scientific, Stansted, United Kingdom), immediately blotted for 5 s, and frozen by plunging in liquid ethane cooled at −180 °C. Grids were then transferred to a Elsa cryo-holder (Ametek SAS, Elancourt, France) and observed at −170 °C. All TEM images were acquired in a Jeol 1400 Transmission Electron Microscope (Jeol, Croissy-sur-Seine, France) operated at 120 keV and equipped with a RIO CMOS camera (Ametek SAS, Elancourt, France).

### 2.6 Fisetin characterization using NMR and LC-MS techniques

Purity of Fisetin raw material and NCs, was verified by using ^1^H and ^13^C NMR spectroscopy and liquid chromatography coupled to high-resolution mass spectrometry (LC-MS). For that purpose, the final product, *i.e.*, the Fisetin NCs suspension, was dried with a vacuum rotavapor, using the same pressure program as for the NCs preparation, then solubilized with convenient organic or water/organic mixture solvent. ^1^H and ^13^C NMR spectra were recorded at 400 MHz, using dimethyl sulfoxide‑*d*_6_ (deuterated DMSO) as a solvent. Correlation Spectroscopy (COSY), Heteronuclear Multiple Bond Correlation (HMBC), and Heteronuclear Single Quantum Coherence (HSQC) analyses were performed for the attribution of the carbon and hydrogen atoms of Fisetin, while Nuclear Overhauser Effect SpectroscopY (NOESY) was performed to apprehend the Fisetin-polymer interactions. ^1^H NMR Fisetin chemical shifts (*δ*) in ppm: 7.92 (1H, d, ^3^J = 9.3 Hz H5), 7.68 (1H, d, ^4^J = 2.2 Hz H2’), 7.54 (1H, dd, ^4^J = 2.2 Hz, and ^3^J = 8.4 Hz H6’), 6.89 (3H, m H6, H8, H5’). The four most deshielded peaks (9.02, 9.25, 9.48, and 10.72 ppm; singlets) belong to the hydroxyl moieties with (1H, s H4’) at 9.02 ppm. ^13^C NMR Fisetin chemical shifts (*δ*) in ppm: 171.9 (C4), 162.2 (C7), 156.2 (C8a), 147.2 (C2), 145.0 (C3’ / C4’), 137.2 (C3), 126.4 (C5), 122.5 (C1’), 119.6 (C6’), 115.5 (C5’), 114.9 (C2’), 114.6 (C6), 114.2 (C4a), 101.8 (C8). LC-MS analysis was performed on an Acquity UPLC system (Waters, Manchester, UK) directly hyphenated to an LTQ orbitrap XL mass spectrometer equipped with an electrospray ionization source (Thermo Fisher Scientific, Bremen, Germany). A BEH C_18_ stationary phase was used (BEH C_18_ 300 Å, 1.7 μm, 2.1 × 150 mm) purchased from Waters, and the mobile phase was composed of milli-Q H_2_O (mobile phase A) and MeOH (mobile phase B), both containing 0.1% v/v formic acid at a flow rate of 100 μL.min^−1^. The mobile phase gradient applied ranged from 10% to 80% B during 38 min followed by 80% B maintained for 3 min. The injection volume was 10 μL. MS analysis was performed in negative ionization mode over 150–2000 range. ESI source parameters were set as follows: ESI voltage +5.0 kV, normalized sheath gas flow rate value was set to 40, and an auxiliary gas flow rate value of 12. ESI nebulizer temperature was set to 300 °C. Capillary voltage and tube lens were set to −35 V and − 150 V. In order to perform Fisetin final product purity assessment after the NCs formulation, the samples were submitted to filtration using 3 kDa Amicon centrifugal filters (Merck Millipore, Molsheim, France) prior to LC-MS analysis.

### 2.7 Effect of cryoprotection on particle size and morphology

Mannitol was used as a protectant to verify its effectiveness in maintaining the physical properties of Fisetin NCs during freeze-drying. At first, NCs suspensions were prepared at three different mannitol concentrations: 0%, 5% and 10% w/v. Then, the samples were frozen at −80 °C. After that, the samples were thawed gradually from −80 to −20 °C, then from −20 to 5 °C, and finally to RT. Then, the size and polydispersity were measured by DLS.

### 2.8 Fourier Transform Infra-Red (FTIR) Spectroscopy

FTIR experiments were performed to determine the structural changes of samples due to chemical interactions by identifying functional groups of Fisetin and P407 raw powders, as well as Fisetin NCs using a FTIR spectroscopy from Shimadzu (Nakagyo-ku, Kyoto, Japan). The FTIR spectra of the samples were obtained in the transmittance mode over a scan range of 4000 cm^−1^ to 400 cm^−1^. 45 spectral scans were conducted on each sample with a 4 cm^−1^ resolution. Notably, pure water or air FTIR signal was subtracted from the signal measured for NCs suspensions or pharmaceutical powders, respectively.

### 2.9 Fluorescence property

To determine if Fisetin fluorescence was maintained after nanocrystallization, fluorescence property was verified by confocal laser scanning microscope (TCS SP8, Leica Microsystems, Nanterre, France) and fluorescence spectroscopy (EL06013039, Cary Eclipse, United States). For the confocal experiments, a drop of the Fisetin NCs suspension was put on a glass slide and cover-slipped. Images were taken with a PL APO x63/1.4 oil-objective with excitation at 405 nm and emission in the 500–550 nm range. For the UV-fluorescence spectroscopy, 1 mL of Fisetin NCs suspension was added into a quartz cuvette to record the fluorescence emission (excitation wavelength: 360 nm) and excitation (emission wavelength: 518 nm) spectra. Noteworthily, the Fisetin NCs suspensions were filtered through a 10-kDa filter at 13500 rpm for 5 min to check the emission signal of the filtrate.

### 2.10 HPLC Fisetin quantification

HPLC experiments were conducted using the DGU-20A3R device from Shimadzu, Kyoto, Japan, to quantify the Fisetin content in the prepared NCs suspensions, which allowed determining their production yield and monitoring the dialysis experiments (*cf.* dissolution study described below). For that purpose, a HPLC column Nucleodur® (endcapped 100–5 C_18_, 5 μm, 250 × 4.6 mm, Düren, Germany) was chosen as the stationary phase, and the optimal mobile phase was selected as follows: Eluents A and B were 2% acetic acid in Milli-Q water, and 0.1% TFA in acetonitrile, respectively. A binary gradient method was used with a 1 mL.min^−1^ total flow rate. The elution gradient started with 15% B for 3 min. From 3 to 6 min, the percentage of eluent B was increased to achieve 50% which was maintained for 6 min, then turned back to 15% for 2 min, followed by an equilibrium stage for 2 min. Under the excitation wavelength of 360 nm, the calibration curve, representing the area under the curve (AUC) as a function of the drug concentration, was established from 17.5 to 350 μM of Fisetin (Fig. S1 and Table S1 of the Supplementary Material). Between each measurement, pure MeOH was injected after cleaning the column with the mobile phase to check the chromatogram baseline. To determine the concentration of Fisetin in NCs preparations, the NCs aqueous nanosuspension was totally dissolved in MeOH (1:9 v/v NCs:MeOH) and then filtered employing a 10-kDa filter for 10 min at 13500 rpm in order to remove the P407 polymer (mean Mw = 13,429.2 g.mol^−1^). Finally, the filtered solution was 10-fold diluted, and 20 μL of this dilution was injected into the HPLC instrument under the above conditions to determine the related concentration using the calibration curve.

The determination of Fisetin encapsulation yield was acquired as follows:

(1) $$\mathrm{Percent encapsulation yield}=\frac{m_{f}}{m_{i}}\times 100\%$$

with, *m*_*f*_ standing for the mass of Fisetin determined from the proceed Fisetin NCs formulations, and *m*_*i*_ the mass of raw Fisetin used to formulate the related preparation.

### 2.11 Dissolution kinetics of Fisetin NCs

*In vitro* release of Fisetin NCs was assessed by the dialysis bag diffusion technique. 1.5 mL of the NCs suspension or raw Fisetin in presence of P407 with equivalent concentrations were placed in a cellulose dialysis cassette (Slide-A-Lyzer™ G2, 10-kDa molecular weight cut off, Thermo Fisher Scientific Inc., Waltham, Massachusetts, United States). Then, the dialysis cassette was immersed, respecting the sink conditions (Lombardo et al., 2021; Phillips et al., 2012), in a compartment containing 290 mL of HEPES buffered saline (HBS) medium (20 and 150 mM HEPES and NaCl, respectively), pH 7.4. Indeed, based on Fisetin solubility (10.45 μg.mL^−1^) at physiological pH and 37 °C, the volume of buffer needed to solubilize this API at the same concentration as the NCs has been estimated at 58 mL. Consequently, the HBS volume introduced in the release compartment was 5 times higher than 58 mL. The whole system was placed in a shaking incubator (Innova 43, Eppendorf, Montesson, France) at 130 rpm, and maintained at 37 °C for 3 days. The receptor compartment was covered with aluminum foil and parafilm to limit the evaporation of the continuous medium buffer. Aliquots (0.1 mL) were withdrawn from the receptor compartment at several time points from 15 min to 3 days, and the equivalent volume of fresh HBS was added to the continuous medium to maintain its overall volume after each sampling removal. Each aliquot was diluted in 1 mL of MeOH and then analyzed by HPLC, as described above in the HPLC subsection.

### 2.12 *In vitro* cytotoxicity on the 3LL and EA.hy926 cell lines

*In vitro* studies were conducted to evaluate the potential of Fisetin NCs on murine cancer and human endothelial cells. EA.hy926 and 3LL cell lines were bought from American Type Culture Collection (ATCC® CRL-2922™ for EA.hy926 cell line, ATCC® CRL-1642™ for 3LL cell line, LGC Standards Ltd., Molsheim, France) and cultured at 37 °C in a 5% CO_2_-humidified atmosphere in the DMEM completed medium. Equivalent Fisetin, poloxamer P407, and DMSO/water ratio concentrations were prepared for reference/control groups. The 3-(4,5-dimethylthiazol-2-yl)-2,5-diphenyltetrazolium bromide (MTT) test was performed as follow. Firstly, 100 μL of cells, at the concentration of 100,000 cells per mL, were seeded in 96 well-plates for 24 h. Secondly, 100 μL of test product was added to each well plate from 1 μM to 60 μM with serial 2-time fold dilutions. Finally, after incubation for 24-, and 72-h, respectively, at 37 °C under a 5% CO_2_-humidified atmosphere, the cell morphology was observed under an inverted microscope. Later on, the medium in each well was removed and replaced by a MTT solution in culture medium (0.5 mg.mL^−1^, 100 μL, M5655 Merck KGaA, Darmstadt, Germany) and incubated for 4 h. To assess the living cells' activity, the culture medium was removed and 100 μL of DMSO were added to each well plate (shaking at 150 rpm for 5 min). The absorbance was measured at 560 nm using a microplate reader (Infinite F200 PRO, Tecan, Männedorf, Switzerland). The results were plotted as a percentage of viable cells as a function of the concentration of the incubated compound, calculated with the equation below.

(2) $$Pe\mathrm{rcent viability}=\frac{A_{560}\;\left(\mathrm{cell plate}\right)}{A_{560}\left(\mathrm{control cell plate}\right)}\times 100\%$$

with, *A*_*560*_ standing for the absorbance measured at 560 nm. DMSO and poloxamer P407 were chosen as control conditions for the free Fisetin and Fisetin NCs, respectively.

The maximal inhibitory concentration for 50% (IC50), and 85% (IC85) of cells viability were calculated by using GraphPad Prism version 9 from the percent viability data.

### 2.13 Morphologic effects on 3LL and EA.hy926 cell lines

For the growth monitoring of both 3LL or EA.hy926 cell lines, each one was seeded in a 24-well plate (100,000 cells per mL for each well) and cultured for 24 h at 37 °C under 5% CO_2_. The DMEM was discarded and replaced by either Fisetin NCs, free Fisetin, poloxamer P407, or DMSO dispersed/diluted in DMEM to reach 10 or 25 μM Fisetin for the NCs, and free Fisetin systems and the equivalent P407 or DMSO content for the control groups. After 24-h exposure, cells in a representative central region of each well were observed at a magnification of ×320 with a Zeiss Axiovert 135 microscope (Carl Zeiss France, Le Pecq, France).

### 2.14 Immunofluorescence microscopy

3LL (200,000 cells per mL) and EA.hy926 cell lines (100,000 cells per mL) were seeded separately on 24-well plates and cultured for 24 h at 37 °C under a 5% CO_2_ humidified atmosphere. Indeed, after several screenings of 3LL cell concentrations, 200,000 cells per mL proved to be the optimal concentration to obtain convenient fluorescence images. The DMEM was discarded and replaced by either Fisetin NCs, free Fisetin, poloxamer P407, or DMSO dispersed/diluted in DMEM to reach 10 or 25 μM Fisetin for the NCs and free Fisetin systems, and the equivalent poloxamer P407 or DMSO content for the control groups. After 24-h exposure, the cells were fixed with 4% paraformaldehyde (10 min at RT), permeabilized with 3% BSA solution containing 0.1% Triton×100 (1 h at RT), and saturated with 2 mg.mL^−1^ of sodium borohydride (10 min, at RT). The cells were then treated with indirect immunofluorescence as follows: cells were incubated with the anti-tubulin primary antibody (1/2000 dilution of the Sigma AB3201 sample, dark for 1 h at 37 °C) and further incubated with secondary anti-rabbit IgG (Fc specific)/fluorescein isothiocyanate (FITC) at 1/400 dilution (Sigma SAB3700846, dark for 45 min at RT). The nuclear counterstaining is conducted by using 4′,6-Diamidino-2-phenylindole dihydrochloride (DAPI) at 1/7000 dilution (Sigma, dark for 30 min at RT). After mounting treatment with Mowiol® medium (Epredia™ from Thermo Fisher Scientific Inc., Waltham, Massachusetts, United State), micrographs were obtained by using a Zeiss fluorescence microscope and a Zeiss LSM-510 confocal microscope (FITC: excitation 488 nm, emission 530 nm; Carl Zeiss France, Le Pecq, France). Referring to previous research (Renault-Mahieux et al., 2021; Touil et al., 2009), the morphological assessment was evaluated thanks to parameters such as circularity and form factor, determined by the help of invert images analyzed with Fiji software, using the following equation:

(3) $$\mathrm{Circularity}=4\pi \times \mathrm{area}\times {\left(\mathrm{perimeter}\right)}^{-2}$$

(4) $$\mathrm{Form factor}=1-\left(\text{circularity}\right)$$

The results were presented as a percentage of the control group using the following equation:

(5) $$\left(1-\frac{\mathrm{treated cells circularity}\;}{\mathrm{control cells circularity}\;}\;\right)\times 100$$

### 2.15 Quantitative evaluation of apoptosis by flow cytometry

The apoptosis assay was carried out using the Annexin V FITC-Apoptosis Detection Kit (eBioscience™, San Diego, California, USA). Briefly, EA.hy926 and 3LL cell lines were seeded onto 24-well plates at densities of 500,000 cells/well and 200,000 cells/well, respectively. After incubation for 24 h at 37 °C, under 5% CO_2_, the cells were exposed to 50 μM of Fisetin NCs, 50 μM of free Fisetin, DMSO, and P407 for 24 h, respectively. Untreated cells were grown as negative control groups. The supernatant was removed and then harvested with 250 μL/well of Trypsin-EDTA (0.05%), followed by centrifugation at 2000 rpm for 5 min. Annexin V-FITC and Propidium Iodide (were equally mixed v:v into the binding buffer (Apoptosis Detection Kit, eBioscience™, San Diego, California, USA) and kept in the dark for further use. All the samples were incubated with 110 μL of the above mixture for 15 min and the reactions were stopped by adding 300 μL of binding buffer. The cells were then analyzed by using Guava EasyCyte™ flow cytometry (Merck Millipore, bioscience, Guyancourt, France).

### 2.16 Angiogenesis assessment

The capillary tube formation was evaluated by *in vitro* angiogenesis assay. 50 μL of growth factor reduced Matrigel® solution (Catalog #354230, Corning®, Bedford, United States) was added to a 96-well plate and incubated for 30 min at 37 °C under 5% CO_2_. For that purpose, the EA.hy 926 endothelial cells (100,000 cells per mL) were suspended in 100 μL of serum-free medium with basic fibroblast growth factor (10 ng/mL, Catalog #610072, BD Biosciences, San Jose, USA) in the presence of either Fisetin NCs, free Fisetin, P407, or DMSO dispersed/diluted in DMEM to reach an optimized concentration of 10 μM of the API for the NCs and free Fisetin systems and the equivalent P407 or DMSO content, as well as the DMEM only for the control groups. After 24-h exposure, tube number and their length were observed at a magnification of ×100 with a Zeiss Axiovert 135 microscope (Carl Zeiss France, Le Pecq, France) and analyzed using the angiogenesis analyzer program from Fiji software. The data were processed with GraphPad Prism version 9 from the micrograph analysis of three independent experiments.

### 2.17 Statistical and fitting analysis

Statistical analysis was done using GraphPad Prism version 9 with a two-way analysis of variance (ANOVA) with a Bonferroni multiple comparison analysis. For the *in vitro* cytotoxicity studies, the percentage of viability as a function of log (concentration) and the non-linear transformation with a sigmoidal dose-response (variable slope) was applied. Statistical significance was represented by *P < 0.05, **P < 0.01 and ***P < 0.001. The release kinetics profile was fitted with the Allometric1 function from the Origin 2019b software (version 9.65).

## 3 Results and discussion

### 3.1 Formulation and characterization of the Fisetin nanocrystals

The bottom-up process of the solvent/antisolvent precipitation developed in our lab for etoposide NCs production (Martin et al., 2020b) has been first optimized to improve the polydispersity index of the etoposide samples. During the process, water was used as an antisolvent meanwhile MeOH was used as the API solvent. Mostly, optimization of the vacuum decompression process allowed us to obtain etoposide NCs suspension with PDI of 0.2 (results not shown) compared to 0.6 reported in previous work (Martin et al., 2020b). Therefore, the optimized preparation method was transposed to the production of Fisetin NCs (Fig. 1a). As physicochemical properties such as particle size play a key role in the cellular uptake of nanoparticles (Honary and Zahir, 2013), optimized and monodispersed Fisetin NCs formulations were designed with diameter in the nanometer scale range and high homogeneity. Indeed, at d0, size measurements have been determined using two different light scattering devices (*n* = 3 for each device used), *i.e.* namely Malvern Nanosizer and Cordouan Nano-kin, allowing us to measure a diameter of 148.6 ± 1.1 and 134.9 ± 1.4 nm, respectively, and PDI of 0.17 ± 0.01 and 0.15 ± 0.01, respectively. An example of the size distribution in intensity obtained with DLS for a given Fisetin NCs suspension is given in Fig. 1b. As far as the Nano-Kin results are concerned, they are shown in Fig. S2. Noteworthily, the NCs, or at least the crystal nuclei, are formed during the solvent/antisolvent injection, since no NCs were measured in the methanolic solution (Fig. S3).

Size is an important parameter for nanomedicine formulations, however, the yield and impurities verification have to be assessed after the production step. Consequently, HPLC was used to quantify the Fisetin content in the nanosuspensions in order to determine the yield of production, while NMR and LC-MS experiments were performed to detect possible impurities.

**Fig. 2:**
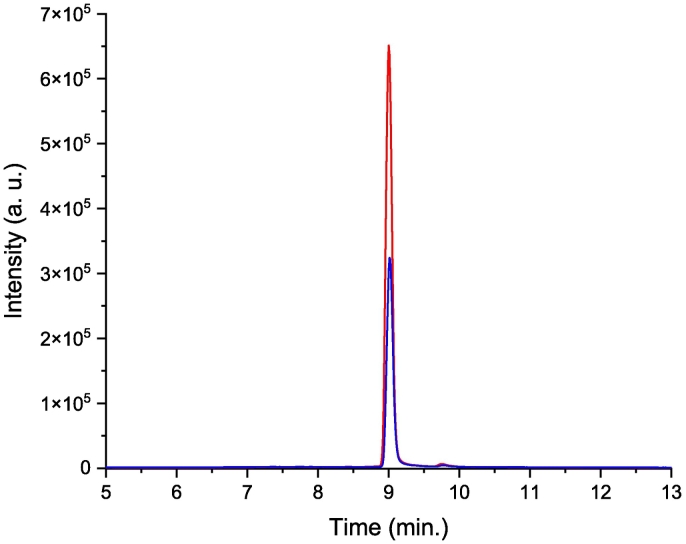
HPLC chromatograms of Fisetin obtained *via* two processes. Raw Fisetin, 64.5 μM (−), and Fisetin/P407 NCs system after dissolution followed by 10-kDa filtration, 144.9 μM (−).

Concerning the HPLC experiments, after screening the optimal mobile phase, experimental parameters were validated (*cf.* Materials and Method part). In these conditions, Fisetin retention time was 9.00 ± 0.01 min (*n* = 10) as shown in Fig. 2.

A calibration curve regarding the AUC as function of Fisetin concentration (y = 21,880 × [Fisetin]) has been established in the 17.5–350 μM concentration range (r^2^ = efficient being 0.9997). For further details, refer to Fig. S1 and Table S1. The limit of detection (LOD) and limit of quantification (LOQ) for Fisetin were found to be 0.052 μM, and 0.16 μM respectively, indicating convenient sensitivity regarding the Fisetin assay for the NCs formulated in the present study (theoretical concentration for 100% yield: 1.456 × 10^3^ μM); and only 4.7 times higher than LOD and LOQ recently obtained by ultra-performance liquid chromatography (Kumar et al., 2021). From the calibration curve equation, the yield of Fisetin in NCs preparations was estimated at 97.0 ± 2.5% (*n* = 5). This high yield of Fisetin NCs is probably due to the non-polar group of the P407 molecule which makes a strong hydrophobic matrix able to interact with Fisetin, allowing its efficient encapsulation. Compared to traditional low molecular weight surfactants, poloxamer P407, a non-ionic triblock copolymer, has a higher solubility and is more selective for heterocyclic and aromatic compounds than aliphatic molecules (Niu et al., 2014). Interestingly, the Fisetin/P407 NCs system presents a high loading content that challenges the already described nanomatrixes such as emulsions, spherulites, and liposomal forms as the latter formulations suffer from non-optimal encapsulation rate of Fisetin, with a mean yield of 58% (Crauste-Manciet et al., 2013; Mignet et al., 2012; Ragelle et al., 2012; Renault-Mahieux et al., 2021; Seguin et al., 2013).

**Fig. 3:**
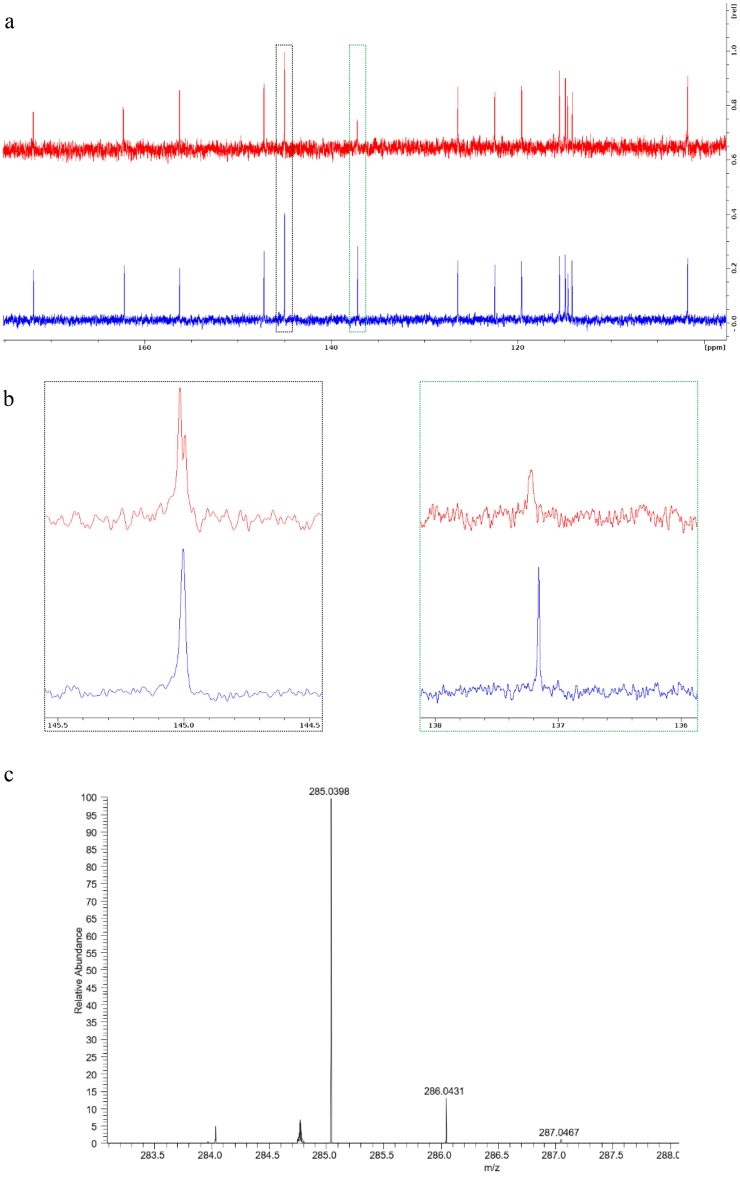
(a) ^13^C NMR spectra obtained from Fisetin NCs/P407 sample (red) and raw Fisetin (blue). (b) Two zooms of the graph region highlighted in panel (a) by doted rectangles. (c) High resolution MS spectrum corresponding to Fisetin obtained from LC-MS analysis of Fisetin NCs/P407 treated sample (theoretical *m*/*z* = 285.0393). (For interpretation of the references to colour in this figure legend, the reader is referred to the web version of this article.)

To get insights into the impact of the nanocrystallization process proposed in the present study, the Fisetin chemical structure at the molecular level has been evaluated from the NCs formulations and compared to the raw API through ^1^H and ^13^C NMR experiments, as well as LC-MS experiments. If we refer to the ^13^C NMR spectrum (Fig. 3a), the 14 peaks obtained for the Fisetin raw material can be also found for the Fisetin/P407 system without any chemical shift. Since Fisetin has 15 carbon atoms (Scheme 1), it can be deduced that two carbon atoms, namely C3’ and C4’, are equivalent for the raw material (one peak at 145.0 ppm). Interestingly, this signal is split into two peaks for the Fisetin/P407 system (Fig. 3b, red curve on the left), indicating that the chemical environment of C3’ differs from that of C4’ when Fisetin is in presence of P407. The previous leads to propose that the interactions between the P407 copolymer and the Fisetin NCs are governed by hydrogen bounds between the two hydroxyls of the catechol moiety and oxygen atoms of poloxamer. The ^1^H NMR data, confirming the same peak position for both P407 and Fisetin/P407 systems, are gathered in Fig. S4. In addition, NOESY experiments have emphasized a dipolar coupling between the hydrogen atom of C2’ and the -CH_2_ moiety of the poloxamer, confirming a spatial vicinity between these two moieties (Fig. S5). Consequently, we propose that the polypropylene glycol (PPG) part the P407 interacts with the phenyl ring of the Fisetin *via* hydrophobic interactions, while the polyethylene glycol (PEG) part interacts with the diol part *via* hydrophilic interactions. It is worth noticing here, however, that the NMR results in the solubilized state may give an idea regarding the possible interactions between poloxamer P407 and Fisetin NCs dispersed in solution. Indeed, if we refer to a previous work, it has been proven that Ketoprofen-PEG interactions deduced from the ^13^C solid-sate NMR experiments were the same as those deduced from the ^13^C liquid NMR experiments performed in dichloromethane (Schachter et al., 2004). Concerning LC-MS analysis of the Fisetin raw material, the MS data obtained demonstrated perfect adequacy with the theoretical information, thus a mass accuracy of 3 ppm could be achieved, and an isotopic profile was in agreement with the atomic composition of the compound. As emphasized in Fig. 3c, the LC-MS analysis of the Fisetin after the NCs formation enabled us to detect and characterize the chemical compound. In this case, as well, high-resolution MS data unambiguously identify non-degradants from the formulated samples.

All data from MS, and ^1^H / ^13^C NMR experiments on Fisetin after nanocrystallization confirm the validity of the process developed. Furthermore, they also prove that the production yield is only impacted by the relative loss of raw material during the different steps of production and not by degradation. Besides, we demonstrated that the drying step after the methanolic injection of Fisetin allows storing the API several days in a lucifungal environment at RT. This result supports the propensity for eventual extemporaneous preparations of Fisetin NCs.

**Fig. 4:**
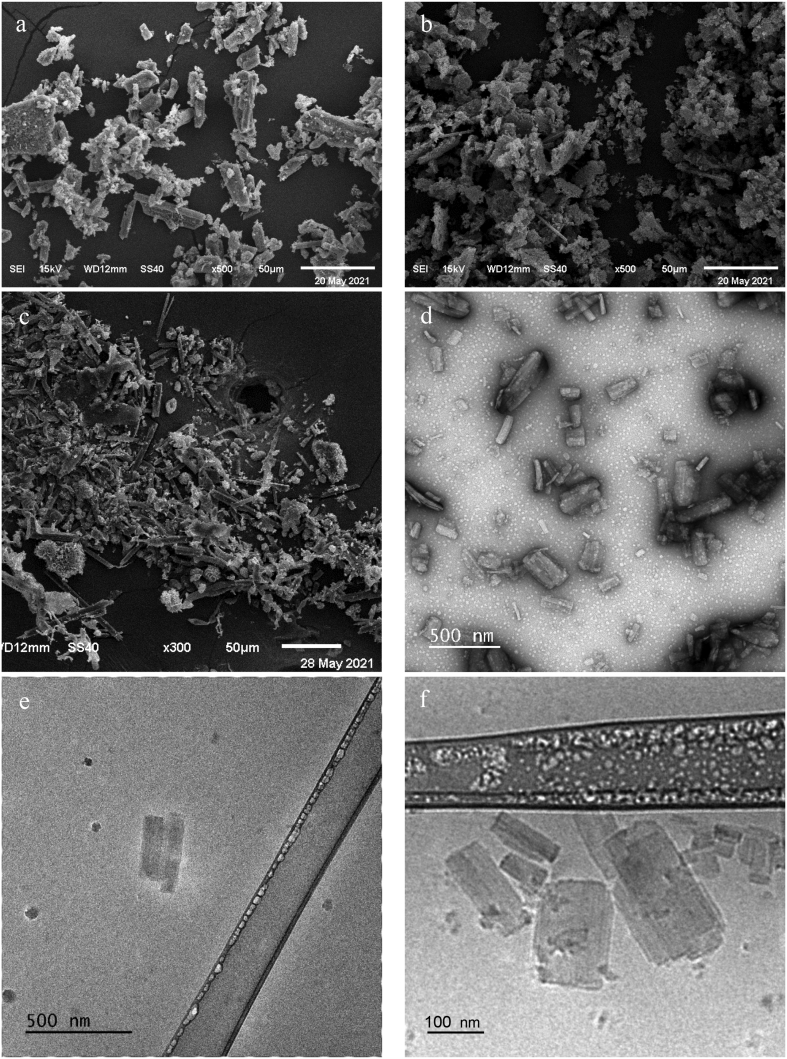
Powder SEM images of raw Fisetin (a), nanocrystals before (b), and after hydration (c) with P407 aqueous solution followed by cryo-lyophilization. TEM (d) and cryo-TEM (e, f) images of Fisetin nanocrystals.

For a deeper understanding of NCs organization in the formulated suspensions, the surface morphology and size of Fisetin particles were identified by different electron microscopy technics for the raw material (SEM, Fig. 4a) and compared to the morphology of Fisetin before (SEM, Fig. 4b) and after (TEM, Fig. 4c and cryo-TEM, Fig. 4e,f) redispersion in P407 aqueous solution.

Raw Fisetin powder particles appear as a rod-crystalline shape with a length of around 50 μm and a width of 5 μm. On the other hand, the Fisetin powder obtained prior to redispersion exhibits agglomerate particles with submicron size, confirming the size reduction of the API during the solvent/antisolvent precipitation process. After water evaporation from the Fisetin NCs suspension, the SEM visualization of the resulting powder demonstrates that the size of the NCs decreases significantly into the nanometer range with the help of the copolymer P407 as a stabilizer; also confirmed by the DLS analysis of Fisetin suspension prepared in the absence of poloxamer P407 (Fig. S3). Nevertheless, due to the agglomeration issue during the freeze-drying process, the accurate size of these freeze-dried Fisetin NCs could not be detected by DLS. Such a difficulty has been well summarized in the literature, specifically in the absence of cryoprotectants (Amis et al., 2020; Li et al., 2009; Liang et al., 2021; Wang et al., 2018). The SEM results and the size measurements were confirmed by TEM experiments performed from the Fisetin NCs suspension dropped on a solid surface at d0 (Fig. 4d). The rectangular crystalline nanoparticles observed present a mean size slightly lower than the mean hydrodynamic parameter measured with the two DLS techniques, as reported elsewhere for other APIs (Kaasalainen et al., 2017; Wilson and Prud'homme, 2021). The discrepancy can be ascribed to the fact that DLS measures the hydrodynamic diameter of the entire nanoparticle, including its surface coating thickness and the associated solvent molecules, while TEM only highlights the nanoparticle core (MacCuspie, 2011; Souza et al., 2016). In addition, cryo-TEM experiments performed with the freeze-dried NCs suspension confirmed the particle size obtained by classic TEM, corroborating the fact that Fisetin NCs shape is not affected by the evaporation of the aqueous environment. This also confirms the nanocrystalline formulation obtained after the hydration step. The NCs obtention was also validated by means of differential scanning calorimetry (DSC) experiments (Fig. S6). Indeed, the melting point of P407 and Fisetin presents a depletion of 26 and 194 °C, respectively, for the NCs powder, while no decrease of the Fisetin melting point has been monitored for the Fisetin/P407 physical mixture. The melting point of physical mixture of P407 decreases by 6 °C. Additionally, the melting enthalpy of P407 in the physical mixture (119.3 J per gram of P407 content) is exactly the same as pure P407 (Martin, 2019). As far as the NCs powder is concerned, the melting enthalpy of P407 is much lower: 4.3 J per gram of P407 content. Thus, the DSC results confirm the nanocrystalline state of the Fisetin and the coating organization of P407, compared to the related physical mixture and Fisetin powder.

**Fig. 5:**
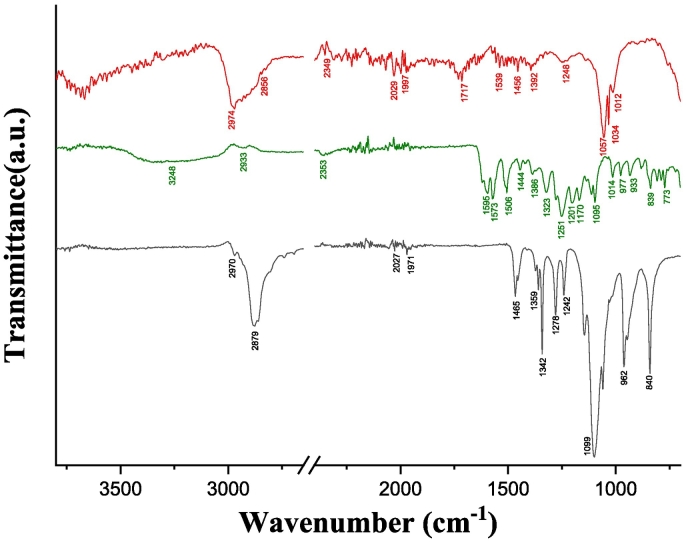
FTIR spectra obtained for Fisetin NCs suspension spectrum (−), Fisetin raw powder (−) and P407 raw powder (−). The intensity of the Fisetin NCs suspension spectrum was 5-fold increased, and the curves were shifted for clarity.

To get insight into specific polymer-NCs interactions, Fisetin raw powder, poloxamer P407 raw powder, and formulated Fisetin NCs were screened by Fourier transform Infra-Red (FTIR) (Fig. 5). The characteristic absorption bands of Fisetin raw powder appear at 3248 cm^−1^ (OH stretching of the hydroxyl groups), 1595 cm^−1^ (C=C stretching of the C3-C2 π-bound), 1573 cm^−1^ and 1506 cm^−1^ (C—C stretching of the C2-C1’ bound), 1444 cm^−1^ (C=C stretching of the aromatic ring), 1251 cm^−1^ (C–O–H bending), 1095 cm^−1^ (C—O stretching), and 839 cm^−1^ (out -of -plane C—H bending). In addition, poloxamer P407 shows principal absorption peaks at 2879 cm^−1^ (C—H stretch aliphatic), 1342 cm^−1^ (in-plane O—H bend), and 1099 cm^−1^ (C—O stretch).

Interestingly, some characteristic absorption peaks of the Fisetin NCs shift compared to free Fisetin from 1573 cm^−1^ to 1539 cm^−1^, and from 1095 cm^−1^ to 1057 cm^−1^, while the two principal absorption peaks of P407 shifted from 2879 cm^−1^ to 2974 cm^−1^, and from 1099 cm^−1^ to 1057 cm^−1^, compared to raw P407. These shifts confirm intermolecular interactions between the poloxamer and the nanocrystallized API. Particularly, the shift of the ν_C–O_ signal for P407 towards lower wavenumbers in the presence of Fisetin NCs, while the aliphatic ν_C–H_ signal shifts towards higher wavenumbers confirms the hypothesis of hydrophobic interactions between Fisetin NCs and the copolymer *via* the aliphatic moieties of the later. These findings correlate with the NMR results described above. Indeed, a chemical shift modification is observed, especially in the hydrogen spectrum domain belonging to Fisetin. Overall, the NMR and FTIR data support the stabilization of the Fisetin NCs by physical coating of the nanoparticles with the copolymer, as it has been also proven for insulin/chitosan or benzoic acid/chitosan nanoparticles (Boonsongrit et al., 2008).

**Fig. 6:**
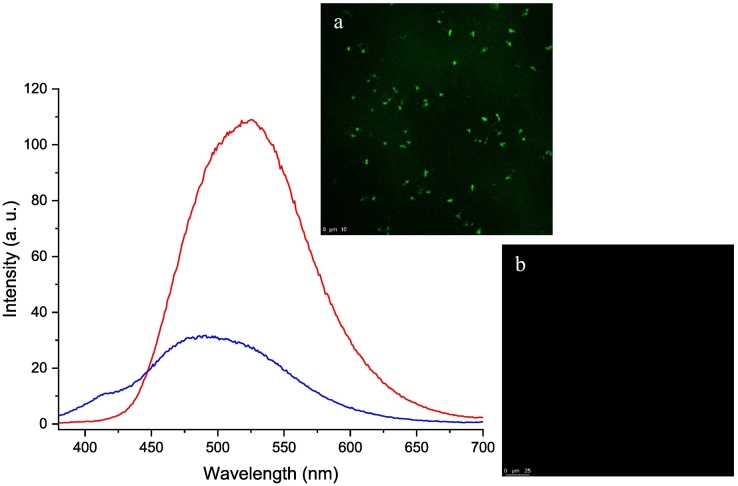
Fluorescence spectra of Fisetin NCs suspension (−), and its filtrate (−) with the corresponding confocal image of Fisetin (insert a), and (insert b), respectively.

Furthermore, the fluorescence property of Fisetin NCs was investigated by excitation at 360 nm (Wang and Zhao, 2016). In Fig. 6a, the maximum intensity fluorescence peak of the NCs suspension is at 528 nm, similar to results reported in the literature upon Fisetin binding to DNA (530 nm) (Sengupta et al., 2015). After filtration on a 10-kDa filter, *i.e.*, after NCs elimination from the continuous medium, one can observe a drastic decrease in the fluorescence intensity combined with a slight hypsochromic shift. Overall, the fluorescence measurements suggest that the prepared Fisetin NCs formulations present aggregation-induced enhanced emission (AIEE) properties (Sengottuvelu et al., 2020). This was also confirmed by confocal microscopy experiments performed on Fisetin NCs suspensions (Fig. 6, insert a).

### 3.2 Dissolution study of Fisetin nanocrystals

**Fig. 7:**
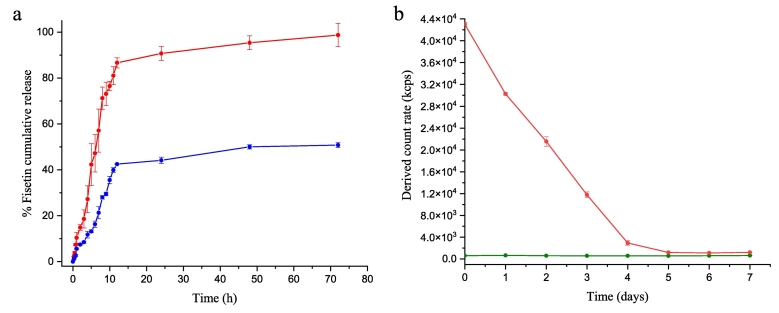
(a) Release kinetics profiles of Fisetin NCs P407 0.083% w/v (red) and raw Fisetin P407 0.083% w/v (blue) in HBS incubated at 37 °C under shaking for 3 days (*n* = 3). (b) Derived count rate performed by DLS for DMEM complete medium containing 10% FBS and 1% penicillin-streptomycin with (red), and without (green) the Fisetin NCs (diluted 7 times). (For interpretation of the references to colour in this figure legend, the reader is referred to the web version of this article.)

Sustained drug release from therapeutic materials is crucial for use in the clinic. The *in vitro* Fisetin release was studied under sink conditions using the dialysis bag tool. The resulting release profiles of Fisetin molecules from the NCs and raw Fisetin / P407 0.083% w/v as a function of the time are displayed in Fig. 7a. For the NCs, a relatively slow and controlled release was monitored for up to 3 days with 98.7 ± 5.1% cumulative release. In contrast, raw Fisetin with P407 0.083% w/v presents lower dissolution rate with 50.8 ± 1.0% cumulative release (Fig. 7b), due to the microparticles (Gigliobianco et al., 2018). Noteworthily, the time needed to completely dissolve in HBS the NCs under sink conditions (Fisetin concentration: 2 μg.mL^−1^) is comparable to the time needed to dissolve in the DMEM medium the majority of NCs (Fisetin concentration: 57 μg.mL^−1^) (Fig. 7). Nevertheless, compared to the NCs free-cell medium, the derived count rate of the diffusion light from the cell medium with NCs dropped from d0 to d4, and stabilized from d5 onwards, suggesting that the NCs may present a slight lower sustained release kinetics in biological fluid.

To confirm that the release profile of the Fisetin NCs was not due to important aggregation within the dialysis cassette, the suspension has been analyzed *via* particle size measurement at d0 and d3 of the dialysis experiments (Table 1). Actually, at d3, a slight increase of the size and polydispersity of NC_S_ were monitored compared to d0, whereas, the raw Fisetin / P407 0.083% w/v formulations exhibited much larger particles size and a complete polydisperse medium.

**Table 1:** Particle size of the Fisetin NCs and raw material with 0.083% w/v P407 before (d0) and after (d3) the dialysis experiments.

		Fisetin NCs	Raw fisetin
d0	Size (nm)	147.9 ± 2.1	846.0 ± 176.3
	PDI	0.14 ± 0.01	0.61 ± 0.16
d3	Size (nm)	244.1 ± 26.2	1749.1 ± 731.2
	PDI	0.35 ± 0.05	0.97 ± 0.11

After determining physicochemical parameters on fresh NCs samples, their stability over time after storage at 5 °C was assessed to estimate the delay of use between production and administration of the final product.

### 3.3 Stability

Table 2 highlights the particle size obtained by diffusion intensity analysis and the polydispersity index of Fisetin NCs measured over time from d0 to d30 using the two light scattering devices detailed in the Experimental Section. The hydrodynamic diameter and PDI of the Fisetin NCs suspensions prepared here showed lower and more homogeneous size than the previous study reported by Dzakwan and co-workers in 2019 with Fisetin NCs stabilized by sodium lauryl sulfate, Tween 80 or Eudragit (Dzakwan et al., 2019). Furthermore, other Fisetin formulations proposed in the literature either as co-crystals with caffeine or nicotinamide (Mohite et al., 2019), as well as other Fisetin nano-formulations presented less optimized parameters compared to the present study (Bothiraja et al., 2014; Dzakwan et al., 2019; Seguin et al., 2013; Wang et al., 2017). Thanks to zeta potential measurement of the NCs formulations (~2 mV. *Cf.* Fig. S7, and Table S2) one can conclude that the stability of the NCs suspension is mainly governed by steric repulsions and not by electrostatic ones (Wang et al., 2020b), which is consistent with the neutrality of poloxamer and Fisetin at the pH of the NCs suspensions (pH = 7.4 ± 0.1). Noteworthily, after NaCl addition in the NCs suspension for zeta potential measurement (final concentration: 10 mM), the nanosuspension presents a similar size compared to Fisetin NCs in pure water (results not shown). Therefore, the above results indicate that the prepared Fisetin NCs present excellent stability, and may have a remarkable potential for overcoming poor drug solubility and bioavailability in the field of targeted drug delivery. This will be later confirmed by the Fisetin NCs stability in biological-like and biological media in the next subsection.

**Table 2:** Particle size stability (in intensity) and PDI (value ± SD) of Fisetin NCs suspension in water, stored at 5 °C, using dynamic light scattering (DLS) and nano-kin analysis, **P < 0.01, *n* = 3.

Time	DLS		Nano-kin	
	*Z*-Ave. (nm)	PDI	Z-Ave. (nm)	PDI
d0	148.6 ± 1.1	0.17 ± 0.01	134.9 ± 1.4	0.15 ± 0.01
d7	150.0 ± 1.0	0.16 ± 0.01	136.7 ± 2.7	0.14 ± 0.02
d14	151.5 ± 1.2	0.16 ± 0.01	142.2 ± 4.0	0.16 ± 0.01
d21	150.3 ± 1.2	0.16 ± 0.01	148.8 ± 2.2	0.16 ± 0.02
d30	150.4 ± 0.9	0.17 ± 0.01	142.3 ± 1.7	0.14 ± 0.02

**Fig. 8:**
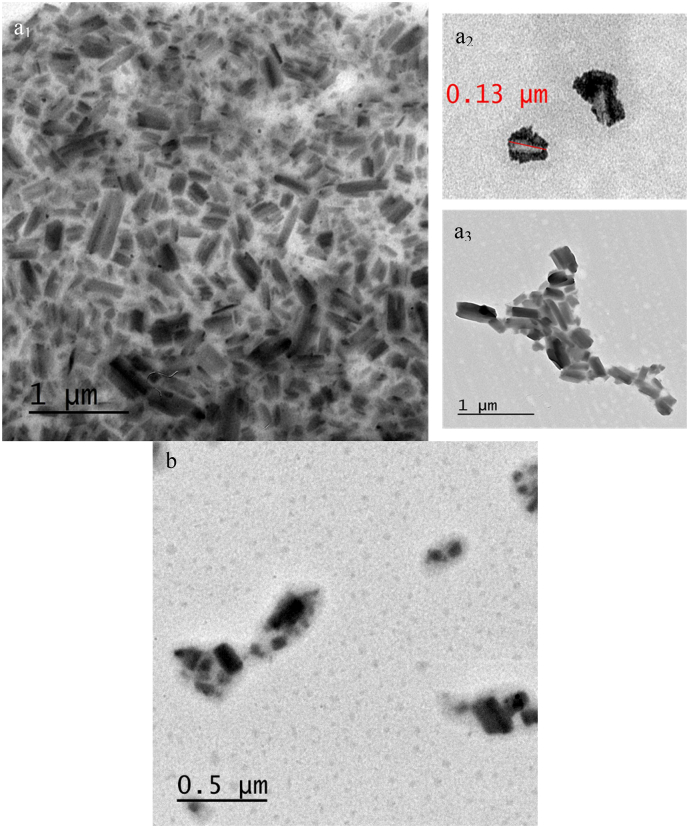
(a) TEM images of Fisetin NCs after 1-month storage at 5 °C. (b) TEM image of Fisetin NCs in 5% w/v mannitol after being frozen and stored for 1 day at −80 °C, then defrosted at 5 °C.

The previous results for the Fisetin NCs suspensions were confirmed by TEM experiments performed after 30 days of storage at 5 °C (Fig. 8). Regarding Fisetin NCs obtained after redispersion in P407 aqueous solution and stored at 5 °C for 1 month, the TEM image reveals that the average diameter is about 130 nm with good stability regarding the rod-shaped and rectangular crystalline with smooth surfaces. As demonstrated in other works (Kaasalainen et al., 2017; Souza et al., 2016; Wilson and Prud'homme, 2021), the size value measured by TEM is slightly lower than that determined by DLS.

### 3.4 Apprehensiveness of the Fisetin NCs cryo-conservation

Cryoprotectants, such as mannitol, trehalose, lactose, and sucrose, can be used during the freezing stages to remove excess moisture, increase long-term storage, and protect the size stability (Almalik et al., 2017; Gokce et al., 2014). In the present study, the effect of mannitol concentration was investigated on the Fisetin NCs to assess storage conditions at −80 °C from d1 to d120 through a freeze-thaw analysis (Table 3). Before freezing, Fisetin NCs suspensions with 0%, 5%, and 10% w/v present the same particle size and PDI data, indicating that the Fisetin NCs are stable in a mannitol environment, as it is the case with NaCl (*cf.* Section 3.3). After being frozen for different times, samples were thawed gradually as detailed in the Experimental Section. Straightforwardly, the particle size of NCs increased owing to the observed aggregation due to a lack of cryoprotectant, which is well highlighted in Table 3. On the contrary, the particle size of Fisetin NCs with cryoprotectant did not increase significantly, which also has been evidenced by the PDI evaluation of the Fisetin NCs containing 5% and 10% w/v mannitol. This could be attributed to the fact that mannitol improved the volume and viscosity of the thawed phase, decreasing the possibility of particles interaction during the freezing process (Amis et al., 2020). Formulations containing 5% w/v mannitol exhibited the best protection, with a relatively small mean particle size of 259.2 nm (PDI of 0.18) after 1 day, and 384.9 nm (PDI of 0.23) after 120 days, so it was selected to continue the subsequent morphology characterization. Besides, ampicillin solid lipid nanoparticles prepared by Alihosseini and coworkers, also showed that 5% mannitol was the optimum choice to prevent particle aggregation (Alihosseini et al., 2015). Morphology evaluation by TEM confirmed the rod-crystalline shape and the lack of significant particle size enlargement of the Fisetin NCs using mannitol as a cryoprotectant agent at 5% w/v after being frozen for 1 day (Fig. 8). In summary, the use of cryoprotectants could reach the desired physical characteristics for prolonged storage effectively, which provides the possibility of overcoming the main hurdle to Fisetin NCs' clinical application.

**Table 3:** Cryopreservation study of the Fisetin NCs. Mean diameter of Fisetin NCs with mannitol as cryoprotectant agent measured by DLS at 25 °C. Comparison with Fisetin NCs prepared in 0%, 5% and 10% w/v mannitol after storage at −80 °C, then thaw at 5 °C before measurement, *n* = 3.

Time after freezing (day)	Fisetin nanocrystal sample					
	no mannitol		mannitol 5%		mannitol 10%	
	Size (nm)	PDI	Size (nm)	PDI	Size (nm)	PDI
0	144.6 ± 5.0	0.16 ± 0.01	153.2 ± 6.2	0.17 ± 0.01	162.4 ± 7.2	0.17 ± 0.02
1	599.5 ± 36.4	0.54 ± 0.1	259.2 ± 8.2	0.18 ± 0.01	278.4 ± 18	0.18 ± 0.03
7	nd (visible aggregation)		248.2 ± 5.6	0.15 ± 0.03	265.7 ± 21.1	0.17 ± 0.02
30	nd		287.1 ± 7.2	0.2 ± 0.01	320.2 ± 27.3	0.18 ± 0.01
60	nd		338.7 ± 12.6	0.21 ± 0.03	359.8 ± 7.7	0.2 ± 0.05
90	nd		345.6 ± 26.8	0.22 ± 0.04	395.1 ± 13.2	0.21 ± 0.02
120	nd		384.9 ± 16.2	0.23 ± 0.02	478.5 ± 9.5	0.27 ± 0.03

### 3.5 *In vitro* cytotoxicity and anticancer efficacy evaluation of the Fisetin nanocrystals

**Fig. 9:**
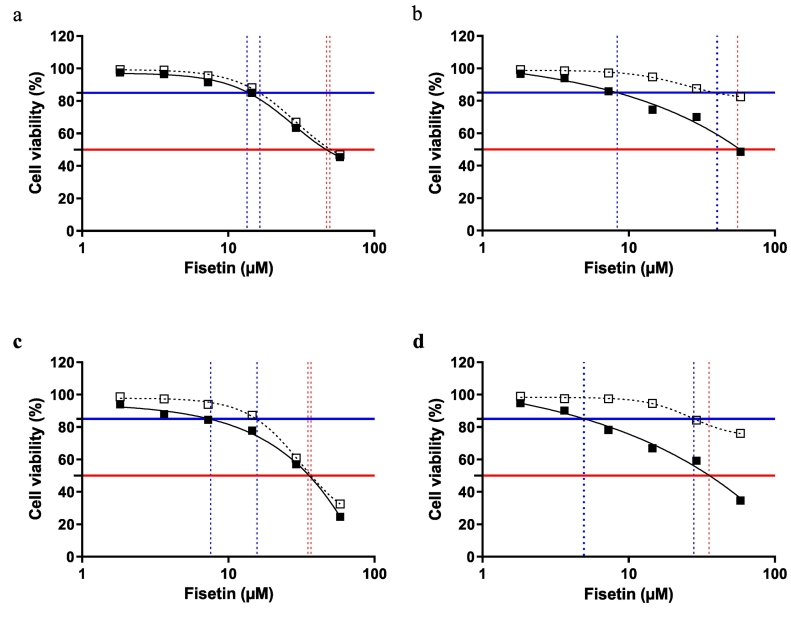
Cell viability results obtained with 3LL cells after 24- (a), and 72-h (c) incubation; and obtained with EA.hy926 endothelial cells after 24- (b), and 72- h (d) incubation. The error bars, obtained from the standard deviation for 3 technical replicates, are smaller than the height of the square data. Data were evaluated by using GraphPad Prism version 9 with a two-way analysis of variance (ANOVA) with a Bonferroni multiple comparison analysis.

To determine whether Fisetin maintains cytotoxicity when formulated into NCs, two different types of cell lines were tested for viability, namely EA.hy926, and 3LL. The percentage of viability curves and inhibition concentrations of 3LL cells depicted in Fig. 9a,c, and Table 4 show no significant difference between free Fisetin and Fisetin NCs. The results are expressed in terms of the API concentration required to kill 50% of the cells (IC50) over the incubation time. The IC50 of free Fisetin incubated for 24 h was 49.6 ± 1.2 μM and 47.0 ± 1.1 μM for NCs, which is consistent with the trend of previous studies (52.4 μM for Fisetin-loaded liposome composed of P90G/cholesterol/DODA-GLY-PEG2000 after 24-h exposure time) (Mignet et al., 2012). In addition, the IC50 decreased slightly, as expected, at 72-h incubation time and was 36.9 ± 0.3 μM and 35.1 ± 0.3 μM for free Fisetin and NCs, respectively. As for Fig. 9b,d and Table 4, the IC50 of Fisetin NCs incubated with EA.hy926 cell lines for 24 h was 55.52 ± 0.41 μM, around three times lower than in recent studies on Fisetin-loaded liposomes composed by DOPC/cholesterol/DODA-GLY-PEG2000 (Renault-Mahieux et al., 2021). Since it has not been possible to determine the IC50 of free Fisetin samples incubated with the EA.hy926 cell line, the IC85 has been calculated to get an idea about the relative inhibition property of the NCs formulations. This property is independent of the incubation time. Nevertheless, these results clearly emphasize the sustained release profile of the Fisetin NCs formulations regarding both 3LL and EA.hy926 cell lines. Interestingly, at given cell viability higher than IC50, one can notice a shift of the Fisetin inhibiting concentration to the high values for free Fisetin dosages compared to NCs formulations for both incubation times and both cell lines, which indicates an improved anticancer effect when the API is formulated as NCs. The effect is more pronounced with the EA.hy926 cell line. As far as the 3LL cell line is concerned, this effect increases with inhibition time.

**Table 4:** The maximal inhibitory concentration in μM for killing 50% (IC50), 85% (IC85) of 3LL and EAhy926 incubated with Fisetin NCs, *p < 0.05, n = 3.

Incubation time	IC	3LL		EA.hy926	
		Free Fisetin	Fisetin NCs	Free Fisetin	Fisetin NCs
24 h	IC85	16.43 ± 0.51	13.45 ± 0.54	40.24 ± 3.95*	8.36 ± 0.32*
	IC50	49.55 ± 1.17	47.04 ± 1.10	–	55.52 ± 0.41*
72 h	IC85	15.70 ± 0.78	7.56 ± 1.06	27.95 ± 1.88	4.94 ± 0.31
	IC50	36.85 ± 0.34	35.05 ± 0.31	–	35.45 ± 0.48

### 3.6 Morphologic effects

**Fig. 10:**
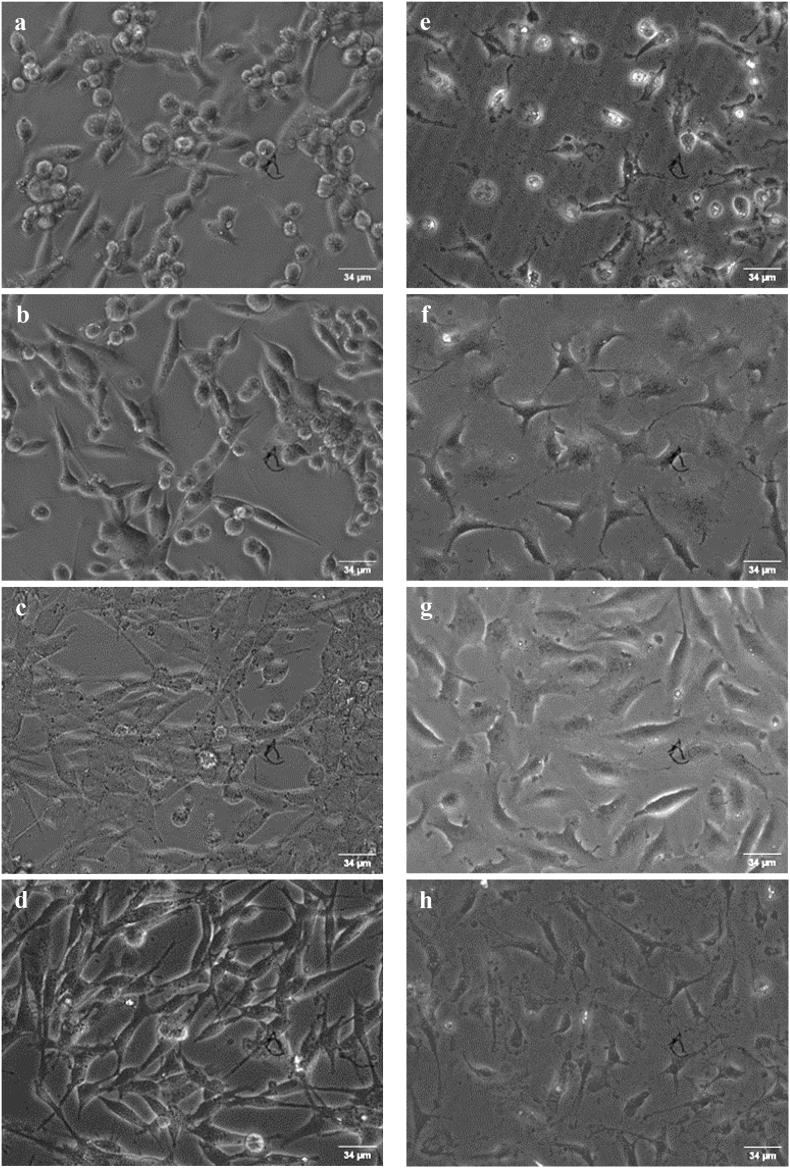
Phase contrast morphology micrographs of 3LL (left column) and EA.hy926 (right column) cell lines after 24-h incubation with 10 and 25 μM Fisetin NCs (a, e), and free Fisetin at the same respective concentration (b, f). Control groups: (c, g) P407, and (d, h) DMSO at the same ratio as the Fisetin NCs and the free Fisetin, respectively.

**Fig. 11:**
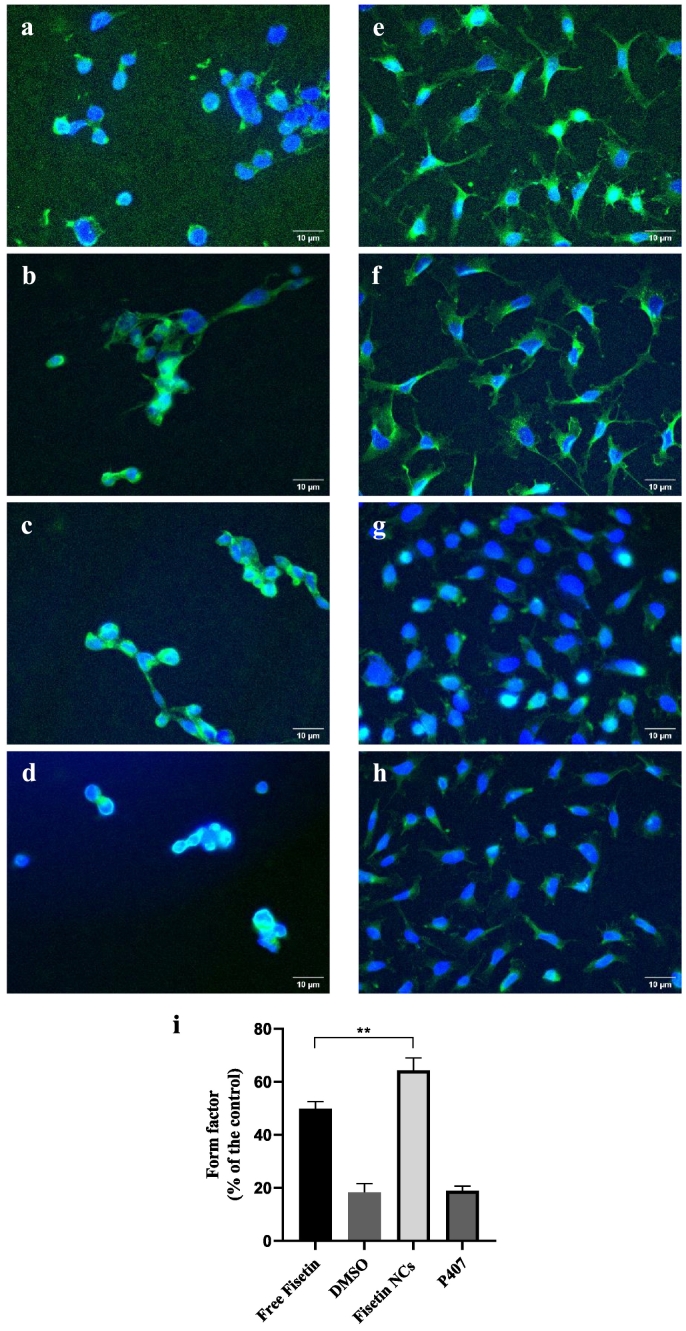
Immunofluorescence micrographs of 3LL (left column) and EA.hy926 cell lines (right column) after 24-h incubation with 10 and 25 μM Fisetin NCs (a, e), and free Fisetin at the same respective concentration (b, f). Control groups: (c, g) P407, and (d, h) DMSO at the same ratio as the Fisetin NCs and the free Fisetin, respectively. FITC (green) and DAPI (blue) indicate the tubulins and nuclei, respectively. (i) The corresponding form factor for each system, calculated as percentage of the control group, *i.e.* the endothelial cells grown in the culture medium only. The data were processed with GraphPad Prism version 9 from immunofluorescence micrographs. Data are mean of triplicate. (For interpretation of the references to colour in this figure legend, the reader is referred to the web version of this article.)

3LL cancer cells and EA.hy926 endothelial cells were used to understand the morphological changes induced by Fisetin NCs to guide future *in vivo* studies. We wanted to explore whether the prepared NCs exhibited the same effects onto microtubules as free Fisetin. Fig. 10, Fig. 11 depict 3LL cancer cells and EA.hy926 endothelial cells after 24-h exposure to Fisetin NCs (control: P407) and free Fisetin (control: DMSO). For 3LL cancer cells, compared with the typical fibroblast phenotype of the control P407 and DMSO groups (Fig. 10c and d, respectively), the morphology of the NC-treated cells changed significantly, with the appearance of rounded dead cell morphology (Fig. 10a). A similar change has been noticed in the free Fisetin group (Fig. 10b) at the same concentration of 10 μM. As expected, EA.hy926 endothelial cells exposed to Fisetin NCs (Fig. 10e) and free Fisetin (Fig. 10f) exhibited some cell extensions in comparison with the P407 (Fig. 10g) and DMSO (Fig. 10h) control endothelial cells.

Furthermore, for a better comprehension of Fisetin toxicity on cells, immunofluorescence experiments were performed, allowing the observation of the nuclei and cells tubulin microfilaments. Some 3LL cells after 24 h-incubation with Fisetin NCs or the free API at the same concentration (Fig. 11a and b, respectively) show slight morphologic changes characterized by elongated membrane pseudopodia, indicating that Fisetin prepared as NCs retains its inherent potency. Indeed, it has been demonstrated that Fisetin increases the stability of the microtubule network by inducing the expression of acetylated α-tubulins, markers of protein stabilization (Touil et al., 2009). This is also confirmed by the control experiments, corresponding to 3LL cells exposed to P407 or DMSO, that show no visible morphological changes (Fig. 11c and d, respectively), similar to the untreated cells (results not shown). Additionally, after 24-h incubation with EA.hy926 endothelial cells, Fisetin NCs or free Fisetin at the same concentration (Fig. 11e and f, respectively) caused significant extension of the cells and numerous filopodia compared to the control P407 or DMSO systems (Fig. 11g and h, respectively). As a control, the immunofluorescence experiment with the Fisetin NCs incubated in the free-cells culture medium was also performed (Fig. S8).

Additionally, the Fiji software was used to contour cells and determine form factors to quantify the morphological changes for each cell group. As observed in the formation of cellular processes, form factors increase as a function of the cell contour irregularity. In the present study, for the control EA.hy926 endothelial cells, the mean form factor was 0.52, which was comparable with previous studies (Renault-Mahieux et al., 2021; Touil et al., 2009). The form factor of Fisetin NCs reached 64.3 ± 4.7%, slightly higher than that of free Fisetin with 49.8 ± 2.7% (Fig. 11i).

Altogether, the morphology results confirm that Fisetin NCs improve the bioactivity of the API. It is worth noting that Fisetin NCs induce a greater effect on EA.hy926 than 3LL cells morphology, which is consistent with the cytotoxicity results presented in Section 3.5. Concordantly, anti-angiogenic effects resulting in morphological extensions of endothelial cells reported for other Fisetin formulations (Mignet et al., 2012; Renault-Mahieux et al., 2021; Touil et al., 2009; Wang et al., 2020a) were also obtained with the nanocrystalline form.

### 3.7 Apoptosis evaluation

**Fig. 12:**
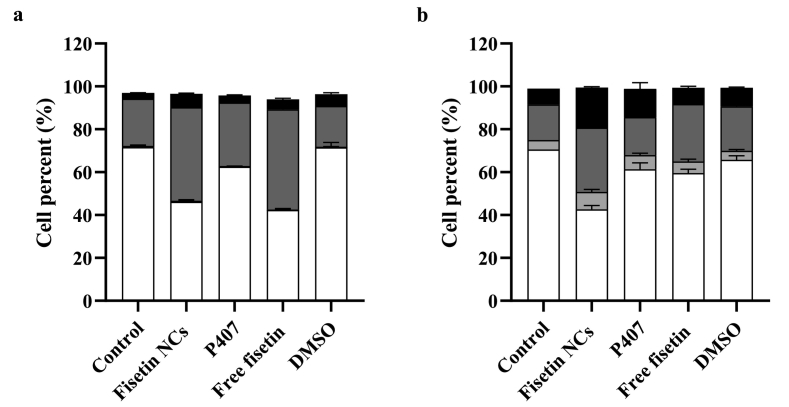
Apoptosis obtained with 3LL cells after 24-h incubation (a); and obtained with EA.hy926 endothelial cells after 24-h incubation (b) with 50 μM Fisetin NCs, free Fisetin, P407, and DMSO at the same respective concentration. Colour code: (black) late apoptosis, (dark gray) early apoptosis, (light gray) necrosis, and (white) viable cells. Control group: untreated cell groups were added to the mixture (Annexin V-FITC, PI and binding buffer). The figures were treated with GraphPad Prism version 9 from flow cytometry device. Data are mean of triplicate (value ± SD).

To determine whether the Fisetin NCs have cytotoxic or anti-proliferation effect *via* induction of apoptosis, 3LL, and EA.hy926 endothelial cells were stained using Annexin V FITC-Apoptosis Detection Kit and analyzed by flow cytometry experiment (Fig. S9). After 24-h incubation, Fig. 12a revealed that Fisetin NCs induced early and late apoptosis stages in approximately 43.6 ± 1.1% and 6.4 ± 0.4% of 3LL cells, respectively, compared to free Fisetin with similar percentages of 46.6 ± 1.6% and 4.7 ± 0.9%, which corresponds to the cell viability and IC50 results studied above.

The effect of Fisetin NCs on the cell percentage distribution of EA.hy926 endothelial cells differs from that of the 3LL cancer cell line (Fig. 12b). In addition to early and late apoptosis, Fisetin NCs and free Fisetin also result in a certain proportion of cell necrosis (8.1 ± 1.2% and 5.4 ± 1.1%, respectively). The percentage of viable cells treated with Fisetin NCs was 42.6 ± 1.8%, whereas the percentage of viable cells treated with free Fisetin was 59.5 ± 1.9%, which implies a higher level of apoptosis (56.9 ± 1.7% and 39.8 ± 1.8%, respectively).

### 3.8 Angiogenesis properties

**Fig. 13:**
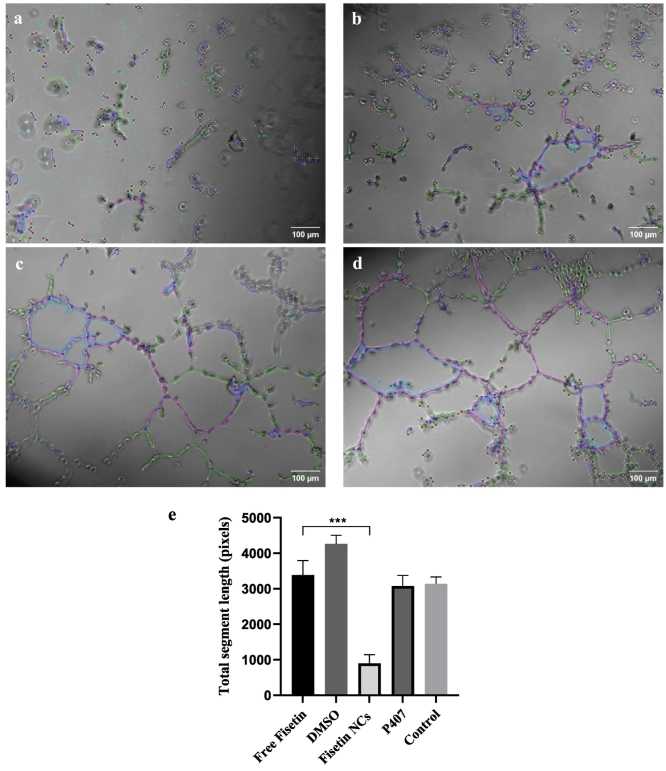
Micrographs of the EA.hy926 endothelial cells cultured in a Matrigel® 3D environment after 24-h incubation with Fisetin NCs (a) and free Fisetin (b) at 10 μM. Control groups: (c) P407, and (d) DMSO at the same ratio. Tubulin organization: (−) branches, (−) segments, and (−) meshes. (e) The corresponding total segment length in pixel for each system, including the control group of the endothelial cells grown only in the culture medium.

EA.hy926 cultured in a Matrigel® environment, *i.e.* an *in vivo*-like 3D gel matrix, allowed following the angiogenic characteristics of endothelial cells *via* the analysis of the branching systems that are related to the angiogenesis process. As shown in Fig. 13, Fisetin NCs significantly inhibit capillary tube formation for endothelial cells compared to the polymer P407 system and, even compared to the free Fisetin system. These results were confirmed by the analysis of the total segment length of the tubulin organization for each micrograph obtained using the angiogenesis analyzer program from Fiji software (Fig. 13e). Fig. S10 details each tubulin system parameter, such as total mesh area, total length, segment number, and branch number. Furthermore, a NCs Fisetin concentration-dependent effect on the tube formation was noticed (results not shown). Therefore, we can highlight the improved anti-angiogenic effect of Fisetin in the nanocrystalline form, an interesting property especially for the treatment of solid tumors, where the accumulation and release of the API in the pathological tissue is essential.

## 4 Conclusion

Nanocrystalline suspensions of Fisetin prepared for the first time in the present study lay out challenging low polydispersity, good colloidal stability and sustained release properties in the frame of nanomedicine development. The Fisetin NCs suspensions were prepared at the nanometer-scale with high yield thanks to an optimized protocol adapted for the active ingredient encapsulation in a copolymer matrix. The nanosuspensions were stable for at least 120 days at −80 °C when stored with a cryoprotectant and 30 days at 5 °C without cryoprotectant. This stability has been proven to be mainly driven by both hydrophobic and hydrophilic interactions between Fisetin and poloxamer P407, especially between the catechol moiety of Fisetin and the poloxamer. A sustained-release profile of the API from the NCs has been demonstrated by drug release experiments allowing considering the potential of this technology to treat different pathologies with optimized administration frequencies, and therefore to improve patient compliance. Furthermore, evaluation of the therapeutic efficiency estimated through *in vitro* experiments on both murine tumor and human endothelial cell lines has shown that Fisetin NCs formulations have the same effect as free Fisetin preparations on 3LL. Additionally, with EA.hy926 endothelial cells, the Fisetin NCs formulations improved therapeutic efficiency by significantly decreasing the IC50 and increasing the apoptosis of the endothelial cells. Furthermore, the *in vitro* model of angiogenesis using EA.hy926 cells exhibits a higher anti-angiogenic effect when the active ingredient is formulated as NCs. These promising results open new anti-angiogenic strategies for treating solid tumors with a natural flavonoid.

## Credit author statement

**Panpan Ma**: Investigation for the nanocrystals production and *in vitro* evaluations, Formal analysis, Writing - Original draft preparation, Visualization. **Johanne Seguin**: Methodology for the *in vitro* experiments, Writing - Review & Editing. **Nhu Ky Ly**: Investigation and optimization of the nanocrystals synthesis, characterization and evaluation. **Luis Castillo Henríquez**: Investigation and optimization of the nanocrystals synthesis, Writing - Review & Editing. **Eva Plansart**: Investigation. **Karim Hammad**: Investigation for the NMR experiments, Interpretation of the NMR data. **Rabah Gahoual**: Investigation for the mass spectrometry experiments. **Hélène Dhôtel**: Investigation for the NMR experiments, Writing - Review & Editing. **Charlotte Izabelle**: Investigation for the electron microscopy experiments. **Bruno Saubamea**: Investigation for the electron microscopy experiments. **Cyrille Richard**: Writing - Review & Editing. **Virginie Escriou**: Investigation for some confocal experiments, Writing - Review & Editing. **Nathalie Mignet**: Writing - Review & Editing. **Yohann Corvis**: Supervision, Project administration, Conceptualization, Methodology, Writing - Review & Editing.

## Declaration of Competing Interest

None.

## Data Availability

Data will be made available on request.

## Appendix A Supplementary data

Supplementary material Image 1
