# Supplementary material for: Designing fisetin nanocrystals for enhanced in cellulo anti-angiogenic and anticancer efficacy
Source: Int J Pharm X. 2022 Nov 9;4:100138. doi: 10.1016/j.ijpx.2022.100138 (PMC9672414; doi:10.1016/j.ijpx.2022.100138)
Supplement: Supplementary file 1 — Supplementary material [file mmc1.docx]

## *Supplementary Material*

# Designing fisetin nanocrystals for enhanced *in cellulo* anti-angiogenic and anticancer efficacy

Panpan Ma ^a^, Johanne Seguin ^a^, Ky-Nhu Ly ^a^, Luis Castillo Henríquez ^a^,
Eva Plansart ^a^, Karim Hammad ^b^, Rabah Gahoual ^a^, Hélène Dhôtel ^a^,
Charlotte Izabelle ^c^, Bruno Saubamea ^c^, Cyrille Richard ^a^, Virginie Escriou ^a^, Nathalie Mignet ^a^, Yohann Corvis ^a,*^

^a^ Université Paris Cité, CNRS, INSERM, UTCBS, Chemical and Biological Technologies for Health Group (utcbs.cnrs.fr), 75006 Paris, France.

^b^ Université Paris Cité, CNRS, CiTCoM, 75006 Paris, France.

^c^ Université Paris Cité, UAR3612 CNRS, US25 INSERM, Cellular and Molecular Imaging Facility, 75006 Paris, France.

* Corresponding Author, E-mail: yohann.corvis@u-paris.fr

**Table S1. Parameters during HPLC test**

| System | UFLC (LC-20AD, UV-detector Shimadzu, Japan) |
| --- | --- |
| Column | C18, 250 × 4.6 mm |
| Method | Binary gradient |
| Mobile phase | A: acetic acid/water (2%/98% v/v), B: Acetonitrile with 0.1% TFA |
| Flow rate | 1 mL/min |
| Wavelength | 360 nm |
| Retention time | 8.9～9.0 min |
| Injection volume | 20µL |

**Table S2. Zeta potential stability of Fisetin NCs after storage at 5 ℃ (n = 3)**

| Time | Zeta potential (mv) |
| --- | --- |
| d0 | -2.0 ± 0.3 |
| d30 | -2.4 ± 0.4 |

**Figure S1.** **HPLC calibration curve of Fisetin solubilized in methanol.**

**
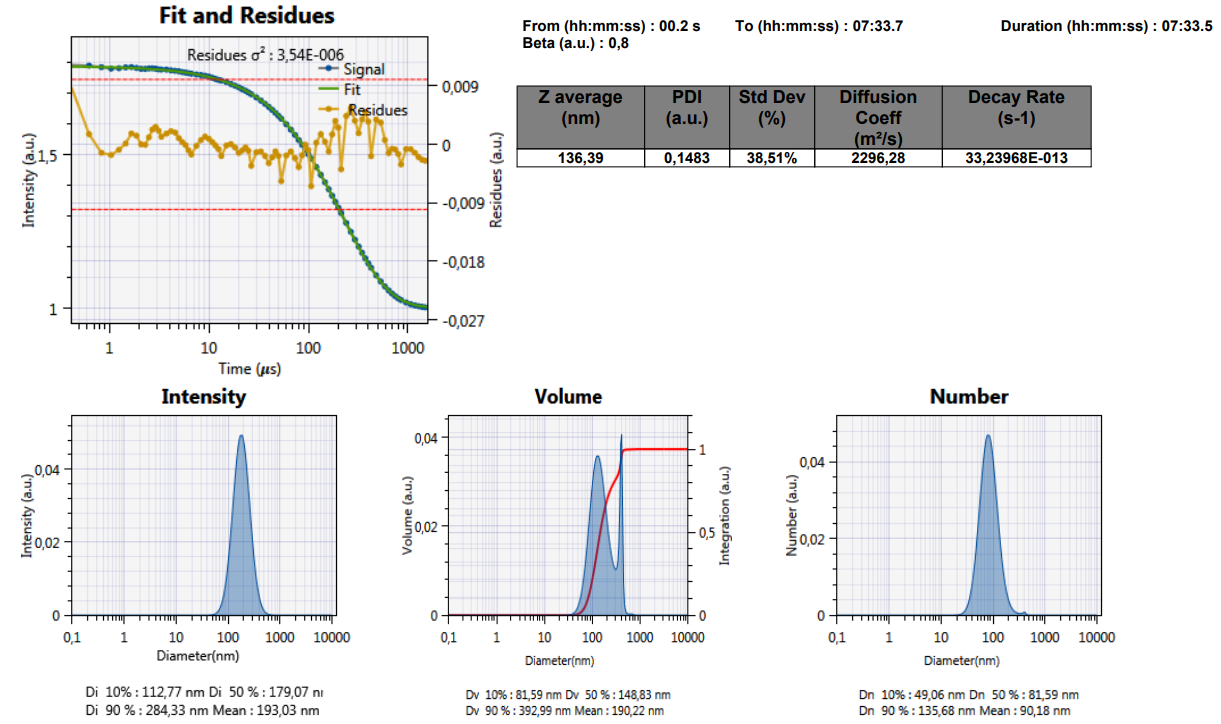
**

**Figure S2. Representative size distribution of fresh Fisetin NCs measured by nano-kin.**

| 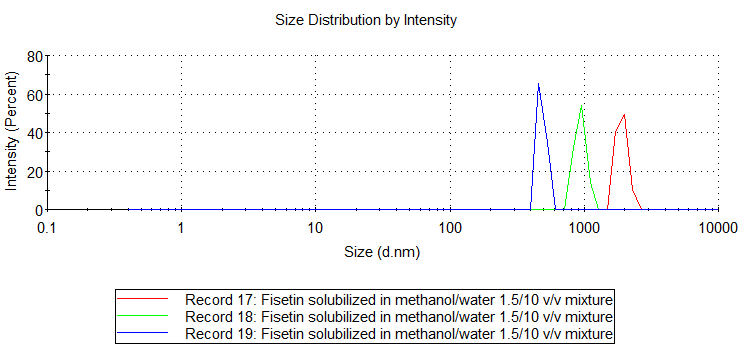  b |
| --- |
| 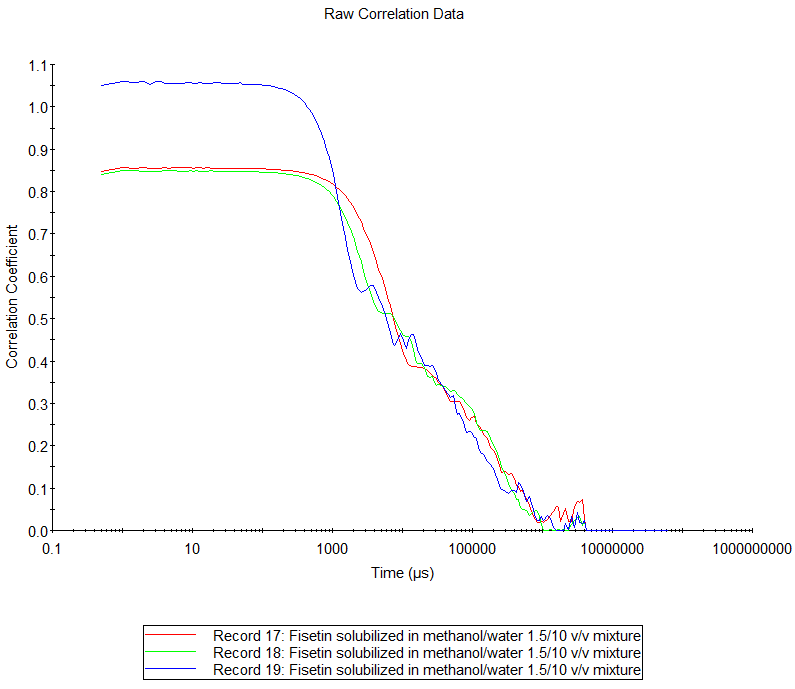  c |
| 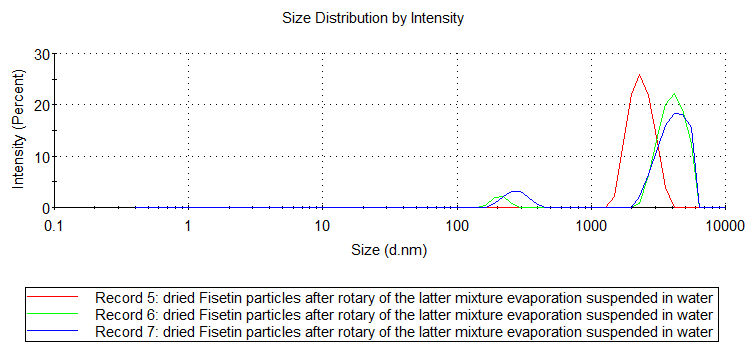  d |
| 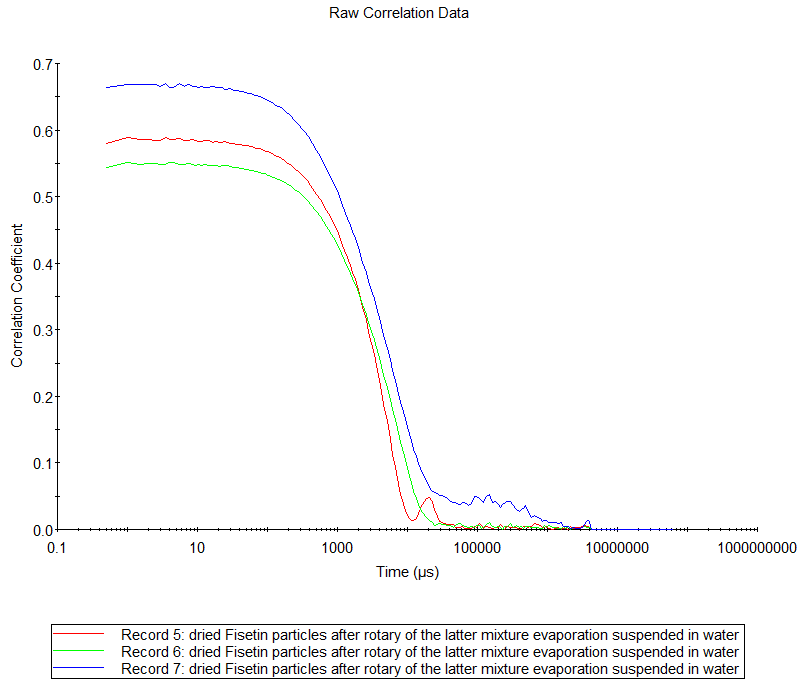 |

a

**Figure S3. Size distribution in intensity (a, c) and the corresponding correlogram (b, d). (a, b) The samples tested were obtained after the methanolic injection of Fisetin in pure water: final methanol/water content of 1.5/10 v/v. The derived count rate, average size and PDI were 11363 kcps, 2718 nm and 0.9, respectively; suggesting big aggregates formation. (c, d) The samples tested were obtained after drying the latter mixture under vacuum rotavapor and rehydrated with pure water. The derived count rate, average size and PDI were 5662 kcps, 2852 nm and 0.31, respectively; suggesting big aggregates formation.**

|   a |
| --- |
|   c  b |
|  |

**Figure S4. ^1^H NMR spectra obtained from Fisetin NCs/P407 treated sample (a), raw Fisetin (b), and raw P407 poloxamer (c).**

**
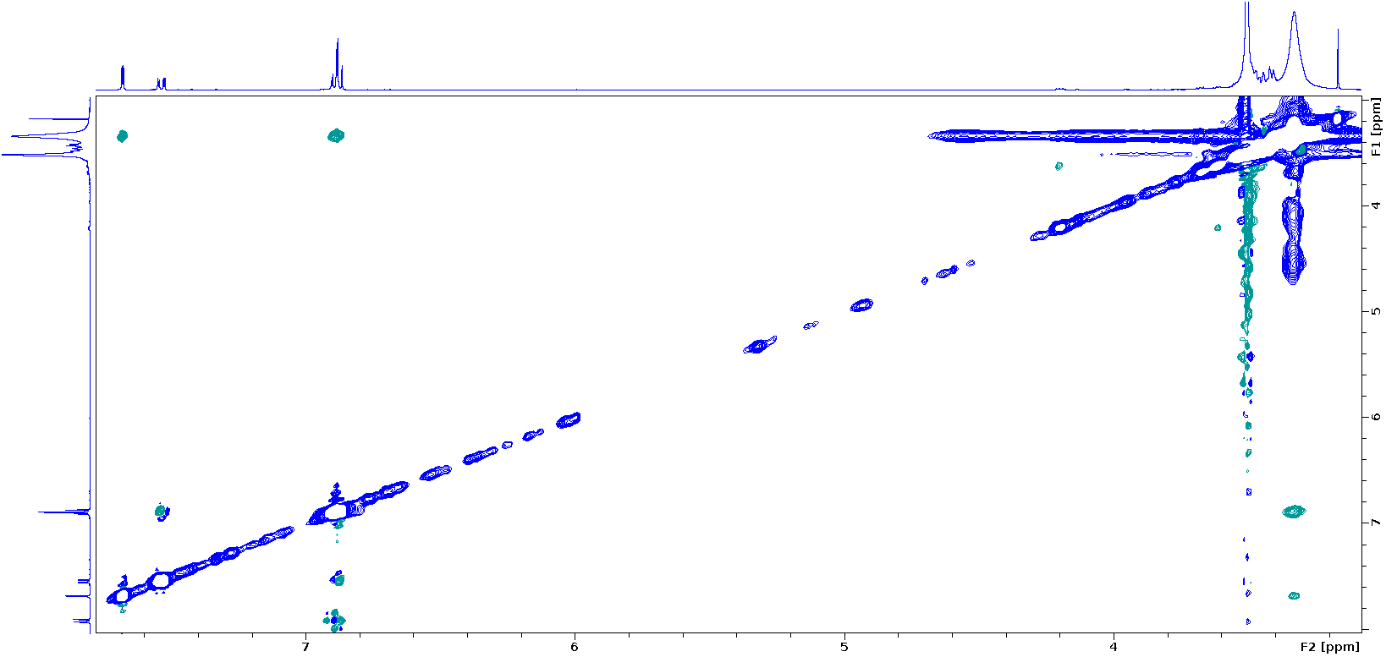
**

**Figure S5. NOESY spectrum for the Fisetin / P407 system. The dotted-line circles indicate a correlation between H2’ of Fisetin and the -CH_2_ moiety of P407.**

**
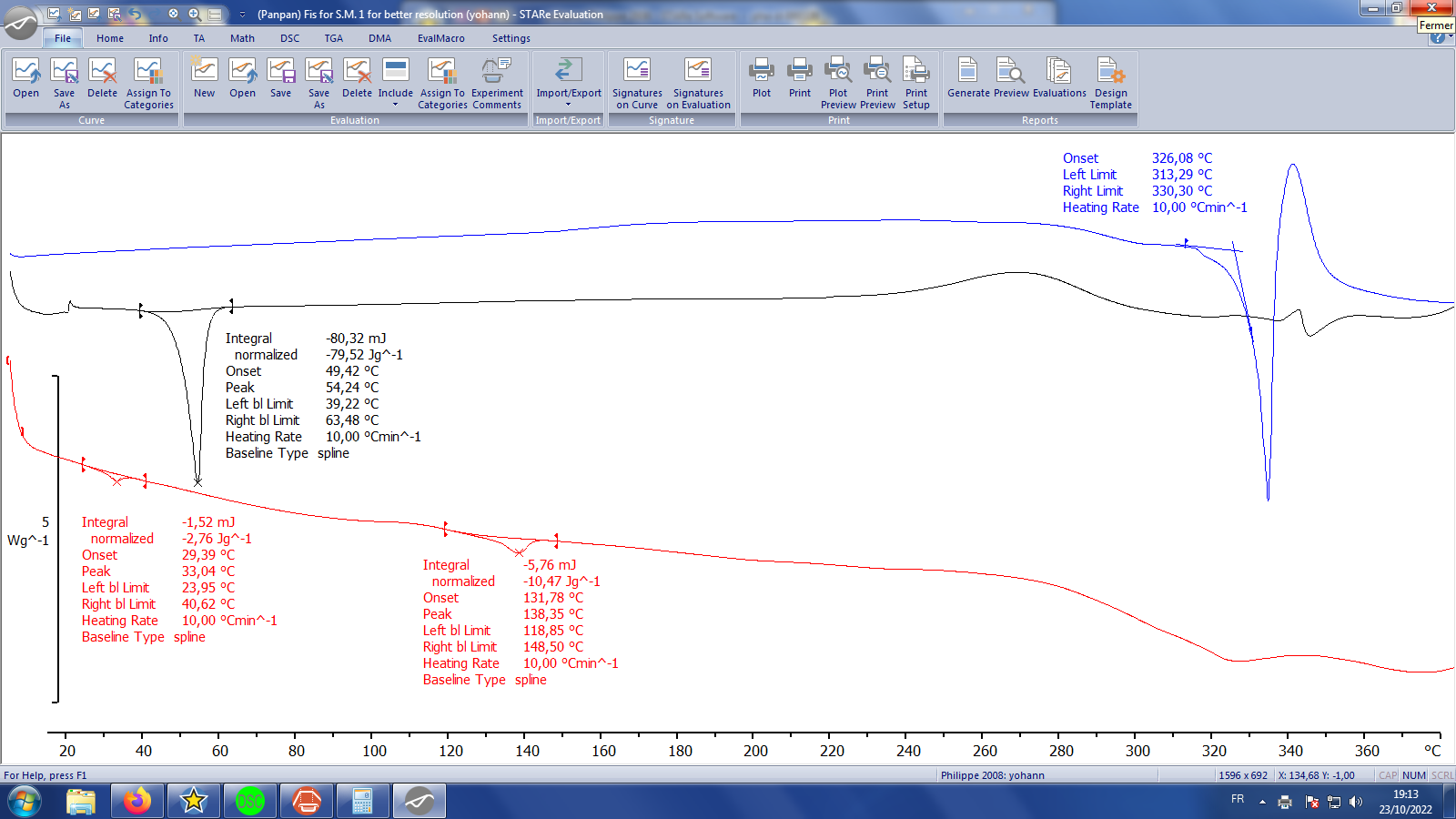
**

Endo

**Figure S6. Mass normalized Differential Scanning Calorimetry (DSC) thermograms obtained for Fisetin microcrystalline powder (blue curve), Fisetin/P407 powder obtained by physical mixture (black curve) and Fisetin/P407 NCs powder obtained by cryo-lyophilization (red curve). The drug/copolymer ratio is the same for the both mixtures. Endothermic transformations give signals pointing down. Each sample was weight in an 40 µL aluminum pan. The pan was then sealed and introduced in DSC1 from Mettler-Toledo (Greifensee, Switzerland), under a dry air flow. Temperature range: from 5 to 400 °C at 10 °C.min^-1^.**


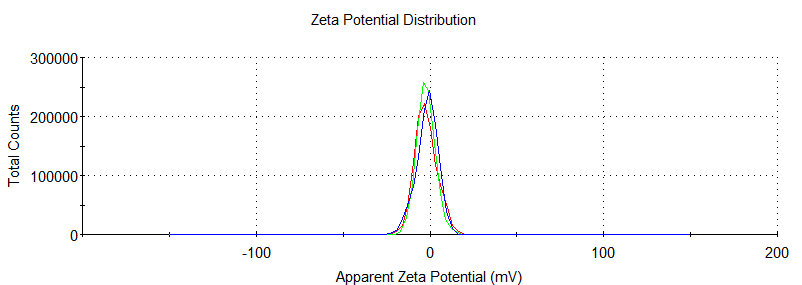


**Figure S7. Zeta potential measurement of Fisetin NCs.**

**
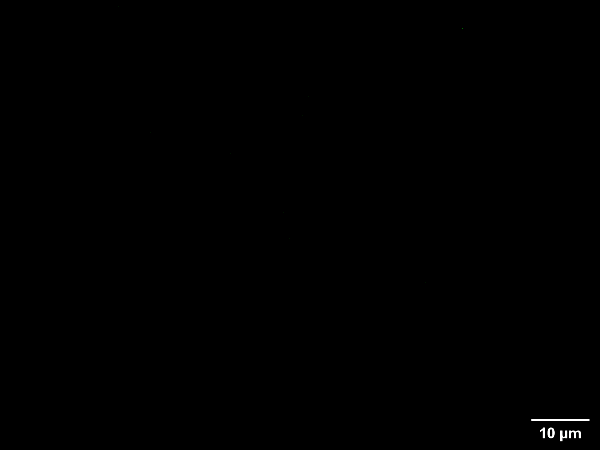
**

**Figure S8. Confocal image obtained for the Fisetin NCs, 25 µM, dispersed in the free-cells culture medium for 24-hour incubation.**

**
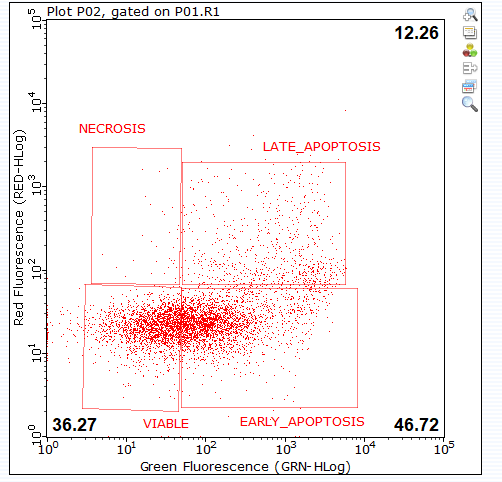
**

**Figure S9. Representative** **apoptosis profiles of EA.hy926 endothelial cells after 24-hour incubation in 50 µM Fisetin NCs obtained directly from the flow cytometry experiment.**

a

b

|   d |  |
| --- | --- |
|   c |  |

**Figure S10. Fisetin NCs inhibit capillary tube formation *in vitro.* ×100** **magnification images of EA.hy926 endothelial cells after 24-hour incubation in 10 µM Fisetin NCs, and free Fisetin. Control groups: P407, and DMSO at the same ratio. The data were processed with GraphPad Prism version 9 from Fiji micrographs, n=1.**
